# CHLOK: a chemigenetic multicolor labeling system to visualize neuronal birthdate and circuit integration

**DOI:** 10.21203/rs.3.rs-7039578/v1

**Published:** 2025-07-08

**Authors:** Giulia Faini, Matthieu Tuffery, Amna Saleem, Lixia Zhang, Felix Du, Guillaume Le Bourdelles, Karine Duroure, Eric Schreiter, Dimitrii Tanese, Valentina Emiliani, Filippo Del Bene, Minoru Koyama

**Affiliations:** 1 Institut de la Vision, Sorbonne Univ., Inserm S968, CNRS UMR7210, Paris, France.; 2 Department of Biological Sciences, University of Toronto Scarborough, Toronto, Canada.; 3 Department of Cell and Systems Biology, University of Toronto, Toronto, Canada.; 4 Janelia Research Campus, Howard Hughes Medical Institute, Ashburn, VA, USA.

## Abstract

Understanding how neurons integrate into developing circuits and contribute to functional activity is essential for decoding brain development and plasticity. However, current methods to study neuronal integration often suffer from low throughput, limited spatiotemporal resolution, or invasive procedures that hinder *in vivo* functional analysis. To overcome these challenges, we present a birthdate-labeling strategy, named CHLOK, based on HaloTag technology and a broad palette of fluorescent synthetic dyes. This approach enables precise multicolor labeling of neurons according to their maturation stage and allows flexible integration into functional assays through compatibility with calcium imaging and optogenetics. We validated CHLOK by mapping birthdate-resolved neuronal activity in the developing visual and motor systems of zebrafish larvae. Our results reveal distinct functional contributions of early- versus late-born neurons, providing new insights into the temporal dynamics of circuit formation. Furthermore, we demonstrate the versatility of this approach, showcasing age-specific multicolor calcium and voltage imaging as well as optogenetic manipulation. By overcoming key limitations of existing techniques, CHLOK offers a powerful, versatile and non-invasive tool for studying neural integration, circuit development and function *in vivo*.

## Introduction

Understanding the developmental trajectory of individual neurons and the maturation of their functional roles within neural circuits is essential to elucidate brain development and plasticity during both embryonic and post-embryonic growth, when progressively more refined functions emerge.

The integration of neurons into developing circuits has been extensively investigated across vertebrate models^1–3^. Newly generated neurons are gradually incorporated into existing circuits by extending axons and dendrites and forming synaptic connections^4^. In several regions of the central nervous system, neurogenesis is spatially organized with newborn neurons being progressively added in topologically distinct areas. Examples include the spinal cord^5^, the hippocampus^6^, the cortex and cerebellum^7^. In this context, the zebrafish larva offers a particularly advantageous model for studying neurogenesis and functional integration due to its optical transparency and rapid development^3,8^. Importantly, a similar topographical organization is observed in the zebrafish nervous system, where some regions exhibit a highly ordered structure characterized by a developmental gradient^9–11^. The most striking example is the optic tectum (OT), the main visual center in the fish brain: neurons generated early in development (earlyborn) occupy central regions, while newly generated neurons (lateborn) are progressively added from peripheral regions throughout the animal’s life^12,13^.

Previous studies were limited by low throughput and methodological constraints, underscoring the need for more advanced tools to examine neurogenesis and functional integration at the population level, ideally in an *in vivo* preparation. Indeed, a comprehensive functional and causal study of neurogenesis and neuronal integration would require a method capable of identifying the maturation stage of individual neurons, and distinguishing earlyborn from lateborn neurons within the intact, developing brain *in vivo*. Furthermore, such an approach should also enable the monitoring of neuronal activity patterns, particularly in relation to specific behaviors or sensory inputs, and permit non-invasive manipulation of neuronal activity. This capability would facilitate precise correlations between neuronal activation, downstream circuit dynamics, and behavioral outcomes.

*Ex vivo* approaches to investigate neurogenesis typically involve the use of Bromo-deoxyuridine (BrdU), Ethyny-deoxyuridine (EdU) or similar Thymidine-analogs, which, when delivered to a tissue, are incorporated into the DNA of cells undergoing replication^14,15^. This allows the identification and analysis of the fate of cells born during the labeling period and all their subsequent offsprings. However, because these approaches require tissue fixation for *post-hoc* staining, they are not applicable for functional studies in living animals.

*In vivo* approaches for tracking individual neurons across development are often based on DNA electroporation in progenitor cells, which sparsely labels one or a few neurons, enabling researchers to monitor their growth, integration, and migration within developing circuits^16,17^. However, this method is inherently low-throughput, labor-intensive, and relies on invasive procedures. Additionally, it has limited scalability for broad circuit investigations. Alternatively, optical approaches provide higher throughput and are less invasive, making them ideal for *in vivo* population studies. Despite the potential of multicolor fluorescent labeling for identifying specific neuronal sub-populations^18,19^, designing strategies for color-dependent birthdate labeling that allow direct access to population-level functional recording and manipulation remains challenging. For example, photoconvertible fluorophores^20^ - which change their emission properties upon exposure to specific wavelengths - allow fluorescent tagging of neurons formed prior photoconversion. However, because the conversion process is binary, it only distinguishes neurons born before or after photoconversion, not those born within a specific time window. More recently, Crispr-based strategies have been developed to activate predetermined sequences of genetic reporters in progenitors undergoing cell division^21,22^. However, they require significant optimization partly due to their complexity and, even under optimal conditions, efficient reporter switching remains limited. Moreover, combining fluorescent age-dependent labeling with functional tools, such as opsins for optogenetics^23^ or calcium indicators for activity monitoring^24^, introduces additional challenges. Indeed, the overlapping excitation and emission spectra of many fluorescent tools result in spectral crosstalks^25^, reducing the accuracy and reliability of both labeling and functional readouts.

To overcome these limitations, we developed a novel chemigenetic approach that provides reliable age-dependent neuronal labeling *in vivo*. This methodology, that we named **CHLOK** for **C**hromatic **H**aloTag **L**abeling for cell **O**ntogeny trac**K**ing, is based on an original framework that exploits the self-labeling HaloTag protein^26^, and its wide range of fluorescent ligands - Janelia Fluor (JF) dyes - known for their excellent photostability and cell permeability^27,28^. The principle of CHLOK is to express HaloTag in differentiating neurons and then load a series of JF dyes with a temporally spaced schedule so that neurons differentiated in each interval get labeled with a distinct JF dye, enabling precise birthdate tagging. After validating this approach against Thymidine-based birthdating, we present a novel set of genetic constructs and labeling strategy to demonstrate its versatility; applications include age-dependent labeling of neuronal processes, simultaneous birthdating of up to five neuronal cohorts, long-term tracking of labelled populations to up to two months, and functional characterization of visual and motor responses in early- and late-born neurons. We also demonstrate the combination of CHLOK with chemigenetic optical tools, including calcium and voltage indicators as well as opsins, enabling direct activity recording and manipulation of specific birthdate-defined neuronal cohorts. Here we applied this methodology to the zebrafish model due to its optical accessibility, but we anticipate its applicability in other genetically tractable systems where JF ligands can be efficiently delivered, such as mouse and *Drosophila*.

Overall, this novel approach addresses key limitations in *in vivo* neuronal birthdating, expands anatomical and functional readouts of emerging neuronal populations and enables their functional manipulation - providing a powerful tool to probe neural development, circuit integration, and plasticity.

## Results

### CHLOK provides a clear view of brain-wide topographical organization based on neuronal age in a living vertebrate.

Upon exposure to its cell-permeable, fast-binding JF dye ligand, neurons genetically-expressing HaloTag become permanently fluorescently labeled, enabling precise visualization of targeted cells^27,29^ or, as recently shown, identification of newly inserted receptors or synaptic proteins^30,31^. In a first set of experiments, we examined if all genetically targeted neurons can be labeled with a JF dye in larval zebrafish using a line co-expressing the HaloTag protein and a membrane-localized GFP. After incubation with a JF dye (here JF_525_) at 5 days post fertilization (dpf) (see Material and Methods), we evaluated the degree of colocalization of the dye and GFP fluorescence, finding 97.43 ± 1.8 % of co-expressing neurons (Supplementary Fig. 1; mean ± s.d, N= 3 fish, 16 planes), confirming a robust and full penetrance of JF dye labeling. We then characterized how well a JF dye can bind to the HaloTag protein across developmental stages and remain fluorescent thereafter, using larvae expressing the nuclear-localized HaloTag under a pan-neuronal promoter (see Material and Methods). Dye incubations were performed at different time points ranging between 0 and 5 dpf and the OT was imaged from 1 to 6 dpf. At all time points, JF dye was successfully incorporated in neurons across the brain, and the labeled neurons remained fluorescent throughout, confirming effective JF dye loading across all developmental stages (Supplementary Fig. 1).

**Figure 1:**
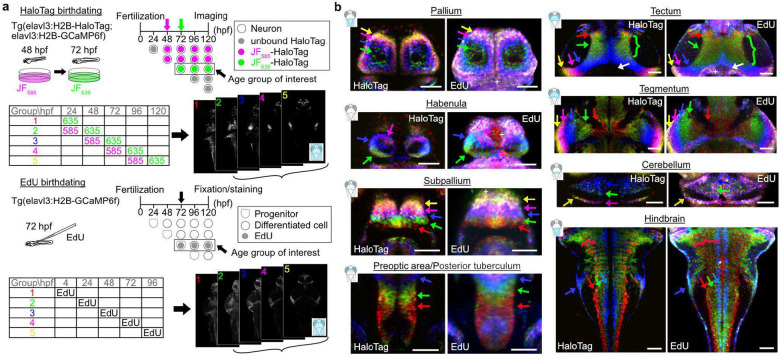
CHLOK and its comparison with EdU birthdating in zebrafish. **a,** HaloTag labeling procedures to label neuronal age groups. Tg(elavl3:H2B-HaloTag) was used to express HaloTag protein in the nuclei of developing neurons. Two JF dyes were loaded in sequence to visualize neurons born in specific time windows. JF_585_: Janelia Fluor 585; JF_635_: Janelia Fluor 635. EdU birthdating procedures: EdU was injected to Tg(elavl3:H2B-GCaMP6f) from 4 to 96 hpf. **b,** Side-by-side comparisons of HaloTag birthdating and EdU staining across the zebrafish brain. The location of the brain region is indicated by a cyan patch overlaid on the fish. Scale bar, 50μm.

Neurogenesis studies often rely on the use of Thymidine analogs such as BrdU or EdU, which are incorporated into the DNA of dividing cells during the S phase^32^. These markers require tissue fixation for post-labeling staining. Here, we set out to compare CHLOK against EdU staining by birthdating cells every 24 hours and compare their distribution at 5 dpf (Fig. 1). To do so, we generated transgenic lines that express the HaloTag protein using the *elavl3* promoter, a well-established marker of early neuronal differentiation^33–35^. To facilitate the direct comparison of the two approaches, we implemented the following strategy (Fig. 1a). First, we added a nuclear localization sequence to the HaloTag to mimic EdU’s nuclear signal. Second, we targeted a single age cohort per animal using two distinct JF dyes, allowing labeling patterns consistent with EdU labeling (neurons in a given cohort were defined as those showing stronger signal from the second dye; see Material and Methods). Third, we registered both datasets to the Zebrafish Brain Browser (ZBB) atlas^36^, using the same transgenic background Tg(elavl3:H2B-GCaMP6f), and generated a 3D volume for each cohort by averaging registered volumes from individual larvae (N ⪴ 4 fish per target). We showed that both approaches revealed spatially clustered age-defined neuronal populations, uncovering topographical organization based on birthdate in more brain regions than previously reported^10,13^ (Fig. 1b). The labeling patterns were largely consistent across the brain, however with a few notable exceptions. First, EdU staining also targeted non-neuronal cell-types, including progenitor cells in the ventricular zone (Fig. 1b, plus signs). Second, the spatial distribution of the EdU-labeled cells appeared broader in the earlyborn populations (Fig. 1b, green and red curly braces in the tectum and the hindbrain, respectively; Supplementary Fig. 2, broad EdU labeling in Groups 1 and 2). These differences are expected as EdU gets incorporated into all dividing cells and their offspring, while CHLOK targets selectively differentiating neurons. Due to this specificity, CHLOK provides a more resolved age-dependent topographical organization in multiple brain regions (Fig. 1b, see habenula and cerebellum), showcasing the inherent advantage of genetic targeting. Having established that CHLOK strategy offers consistent, if not better, birthdating resolution compared to the standard EdU method *in vivo*, we further examined its unique applications.

### CHLOK reveals brain-wide chronological organization at subcellular level and enables simultaneous labeling of multiple neuronal age groups.

One advantage of CHLOK is its ability to visualize specific subcellular compartments of a target neuronal age group by fusing a localization sequence to the HaloTag protein. This is in contrast to the Thymidine-based birthdating approach where the signal is restricted to the nucleus. Here we targeted axon terminals using the synaptophysin tag^37,38^ and somatodendritic processes using the Voltron1 construct containing the HaloTag protein fused with the Kv2.1 localization sequence^39–41^. We used *TgBAC(slc17a6:EGFP)* - a transgenic line expressing EGFP also in the neuropil - as a common background to ensure precise registration of subcellular processes. We targeted the same neuronal age groups as in Fig. 1 and generated the population average for each age group (N ≧ 4 fish per group). The area occupied by each age group was defined by the voxels showing stronger signal from the corresponding target dye (JF_635_ for Groups 2–5 and JF_585_ for Group 1; see Material and Methods). This procedure enables precise visualization of the newly area innervated by the targeted age cohort.

**Figure 2:**
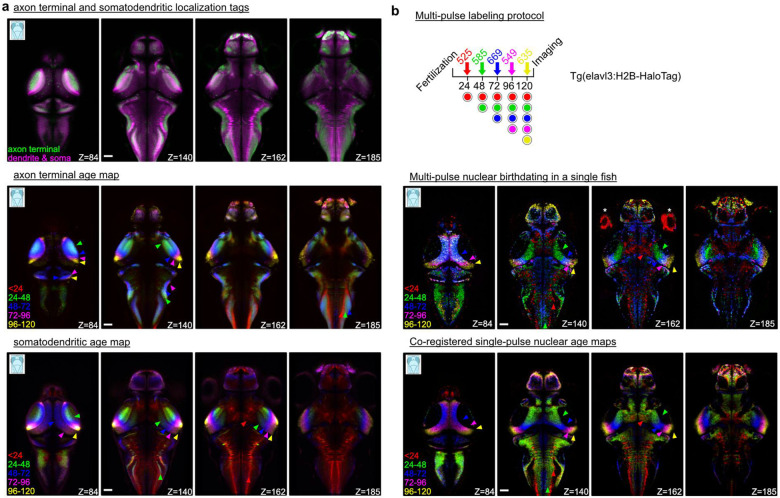
Subcellular visualization of neuronal age groups and simultaneous visualization of multiple age cohorts by CHLOK. **a,** Subcellular visualization of individual age groups. (top) Distribution of HaloTag-JF_585_ localized to axon terminals (green, msypb) and somatodendritic processes (magenta, Kv2.1) in 5 dpf zebrafish. JF_585_ was loaded at 4 dpf (middle). Visualization of new axon terminal areas occupied by each age group. (N ⪴ 4 fish per group). Each age group is color-coded as in Fig. 1. Red: <24 hpf, Green: 24–48 hpf, Blue: 48–72 hpf, Magenta: 72–96 hpf, Yellow: 96–120 hpf (bottom). Visualization of new dendritic areas occupied by each age group (N ⪴ 4 fish per age group). Scale bars: 50μm. **b,** Simultaneous birthdating of multiple age groups in a single fish. (top) Experimental timeline for sequential loading of Janelia Fluor dyes and confocal imaging. 525: JF_525_, 585: JF_585_, 669: JF_669_, 549: JF_549_, 635: JF_635_ (middle). Visualization of five age groups in a single fish. The volume is registered to the ZBB atlas for the ease of comparison. Each age group is color-coded as in Fig. 1. Red: <24 hpf, Green: 24–48 hpf, Blue: 48–72 hpf, Magenta: 72–96 hpf, Yellow: 96–120 hpf (bottom). Co-registered single-pulse nuclear age maps in Fig. 1 are shown as a reference. The Z value indicates the slice position in the ZBB atlas space.

We first confirmed the distinct labeling patterns of the two subcellular localization tags (axon terminals and somatodendritic processes) by comparing the JF_585_ signal in Group 5 which captures the majority of neurons except those born between 4 and 5 dpf (Fig. 2a **top**). Interestingly, this comparison revealed not only the expected differential labeling in the cell body areas but also distinct domains within the neuropil that are dominated by either axonal terminals or dendrites. Furthermore, CHLOK-based labeling of these subcellular tags revealed distinct age-dependent organization within these domains (Fig. 2a). We observed that axon terminals from the later-born populations occupy progressively the outer layer of the neuropil area across multiple brain regions such as the tectum, the cerebellum, and the hindbrain (Fig. 2a **middle**, see the ordering of colored arrowheads). In contrast, the majority of dendritic domains were colored by the earliest-born group (<24, Fig. 2a **bottom**) except the tectum. Closer inspection of JF_585_ and JF_635_ signals across age groups suggests that this pattern reflects the mostly overlapping or smaller dendritic processes of the late-born populations (Supplementary Fig. 3). Taken together, CHLOK labeling of subcellular processes revealed a brainwide chronological organization of axonal and dendritic structures, highlighting a unique advantage of CHLOK to visualize the subcellular architecture of birthdated neurons.

Another advantage of CHLOK is the broad spectrum of JF dyes biocompatible in zebrafish. This characteristic provides an original opportunity to simultaneously visualize multiple neuronal age cohorts in individual fish. To determine whether we could reproduce the nuclear age map using sequential dye loading in a single fish, we applied five distinct JF dyes every 24 hours and imaged the larvae at 5 dpf with a white laser confocal microscope to excite each dye at its optimal wavelength (Fig. 2b **top**, see Material and Methods). We then created a differential map to isolate each age cohort, similar to the population-based map of Fig. 1, to minimize the crosstalk across channels (Fig. 2b **middle**). This approach successfully captured the overall chronological organization of soma positions observed in the population-level dataset (Fig. 2b **bottom**) with some differences in the boundary between age groups. In summary, we demonstrated a unique capability of CHLOK to simultaneously visualize up to five distinct age cohorts in a live animal.

To our knowledge, this CHLOK offers the first *in vivo* age-related labeling, enabling an unprecedented visualization of the topographic organization of neuronal maturity within the same developing animal.

### CHLOK enables persistent, stable and bright labeling across juvenile stages.

**Figure 3:**
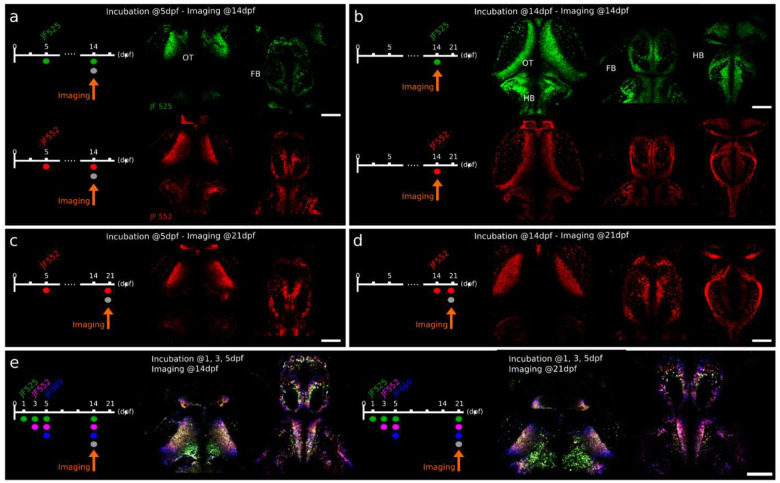
Persistent JF dye labeling in juvenile brains with *CHLOK*. Confocal images showing the distribution of halotag-expressing neruons in zebrafish larvae, with dye incubation and imaging performed at different developmental time points (see corresponding schematics). **a,** 14 dpf larva labeled with JF_525_ (green) or JF_552_ (red) at 5 dpf. **b,** 21 dpf larva labeled with JF_552_ at 5 dpf. **c,** Same as in (a) but in larvae incubated at 14 dpf. **d,** 21 dpf larva labeled with JF_552_ at 21 dpf. **e,** Multilabeled larvae (at 1, 3 and 5 dpf) imaged at 14 dpf (left) or 21 dpf (right). Scale bar: 100μm. OT: optic tectum. HB: hindbrain. FB: forebrain. dpf: day-post-fertilization.

To assess the stability and persistence of the HaloTag labeling, we extended our analysis to juvenile stages. By focusing on fish labeled at larval stages with single or multiple dyes, we tracked neurons well beyond their initial integration into circuits. Interestingly, our results demonstrate that HaloTag-based labeling is effective for visualizing and tracking neurons over extended periods (up to 8 weeks post-fertilization; Supplementary Fig. 4). Specifically, we showed that larvae incubated at 5 dpf with either JF_525_ (green) or JF_552_ (red) retained clear labeling when imaged at 14 or 21 dpf (Fig. 3a,c). Furthermore, the dyes not only persist through later development but can also be incorporated at juvenile stages (*i.e* incubation at 14 dpf), as shown in Fig. 3b,d. Importantly, we performed triple incubations at larval stages (at 1, 3, and 5 dpf) and were still able to detect distinct birthdating labeling when imaging at either 14 dpf or 21 dpf (Fig. 3e), confirming the temporal resolution and persistence of this method. In addition, this stable long-term labeling enabled the identification of labeled neurons in fixed and cryosectioned brains and retinas (Supplementary Fig. 4a), making it compatible with histological analysis. In a final set of experiments, we incubated the larvae at 5, 10 and 14 dpf, and imaged them at 14 dpf (Supplementary Fig. 4b), revealing patterns of **t**opographic neuronal organization in juvenile fish that mirrored those observed during larval stages (Fig 2c). Together, these results highlight the versatility of the HaloTag system for longitudinal studies of neuronal maturation and integration, from early development into juvenile stages.

### CHLOK enables to track the functional contribution of early- and lateborn neurons in visual processing.

To explore the functional integration of early- and late-born neurons, we combined CHLOK birthdate-labeling with visually evoked *in vivo* calcium imaging in the OT (see experimental protocol in Supplementary Fig. 6a).

**Figure 4:**
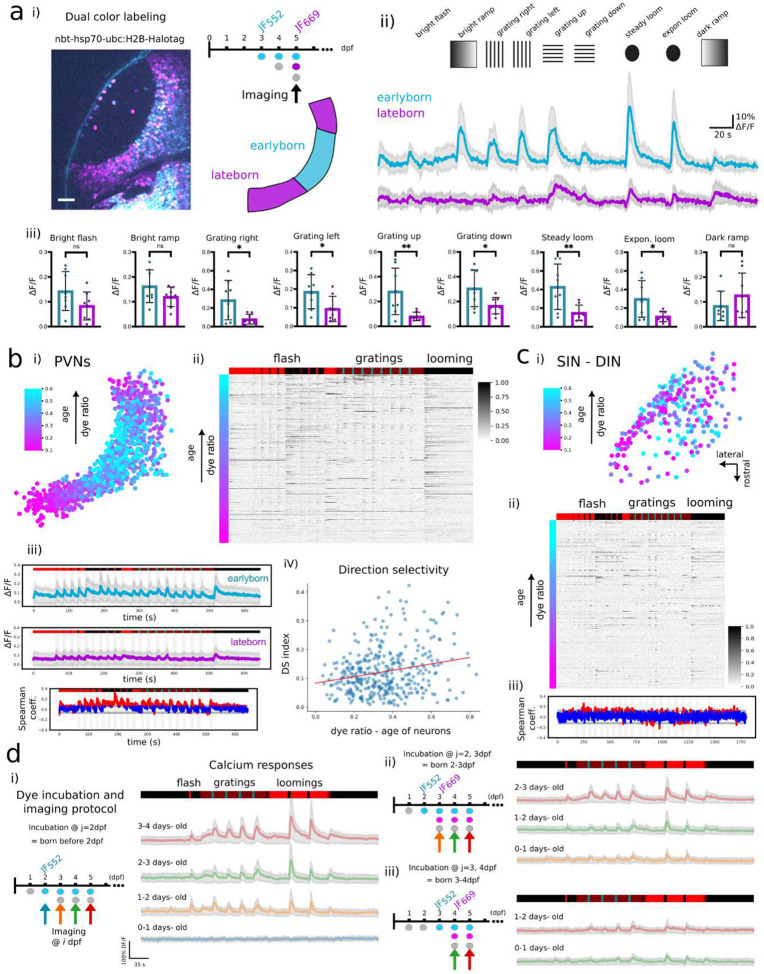
Visual activity of early- and lateborn neurons with CHLOK. **a,** (i) Top: dual-color labeling in *Tg(Xla.Tubb2*-*hsp70-ubc:H2B-Halotag; elavl3:H2B-GCaMP6s)* at 2 different time points during development (3 and 5 dpf). Bottom: Fluorescence dual color images of the larval OT. The larvae also co-express GCaMP6s under a pan-neuronal promoter. More mature neurons are in cyan and lateborn neurons in magenta. The schematic on the right represents the corresponding ROI used to integrate the calcium activity of the two populations. Scale bar: 25 μm. (ii) Representative GCaMp6s calcium traces of early-born (cyan) and lateborn (magenta) neurons in response to a series of visual stimuli (top row), including flashes, gratings, and looming stimuli. GCaMP fluorescence signals were integrated from the regions in (i). Each grey line represents one larva (N = 8 larvae). Colored lines represent the average traces. (iii) Quantification of ΔF/F peak responses between early and lateborn neurons, showing significant differences in response amplitudes for select visual stimuli, particularly moving gratings and looming disks (N = 6 larvae). (iv) Colormap representation of the two-population activity separated across the 8 investigated larvae. **b,** (i) Spatial distribution of PVNs color-coded according to the ratio of fluorescence intensity of the two dyes JF_552_/(JF_552_/JF_669_). Light blue color represents lateborn neurons. (ii) Raster plot of ΔF/F traces from individuals’ cells from (i) neurons of 5 larvae. Larvae were presented with visual stimuli, schematically represented by the upper color bar (red meaning high visual intensity, black indicating dark. See Methods for details). (iii) Top and middle: ΔF/F average traces from the earlyborn (cyan) and lateborn (magenta) subpopulation of cells from (ii). Semi-transparent rectangles indicate timing of visual stimuli. Bottom: Spearman coefficient between dye ratio and ΔF/F, plotted in function of time. In red, sections in which the activity of the two populations show statistically significant differences (see Methods for more details). (iv) Direction selectivity (DS) index in function of the dye ratio of individual neurons (p* =0.03, Spearman coefficient). **c,** Spatial distribution of neurons in the neuropile (SIN and DIN), color coded according to the ratio of fluorescence intensity of the two dyes (N = 9 larvae). Light blue color represents lateborn neurons. **d,** For each subpanel, Left: Schematic of the labeling protocol for either single-dye labeling (JF_552_ at j = 2 dpf; panel i) or dual-dye labeling (JF_552_ and JF_669_ incubated at days j and j+1 dpf, with j = 3 and 4; panels ii and iii, respectively). Functional imaging was performed at i ≥ j dpf during the delivery of visual stimuli sequences (same as in b). Cells labeled only with JF_552_ define populations born either before 2 dpf (i) or between days j and j+1 (ii, iii), and are thus (i – j) days old at the time of imaging. Right: Average calcium responses of these age-defined populations to the visual stimuli sequence, shown for different incubation and imaging time points, corresponding to neuronal populations of varying ages (N = 3–4 larvae). OT: Optic Tectum. PVN: Periventricular Neurons. SIN: Superficial Interneurons. DIN: Deep Interneurons. dpf: day post fertilization.

By crossing the transgenic line *Tg(Xla.Tubb2-hsp70-ubc:h2b-Halotag)* with a pan-neuronal GCaMP6s-expressing line (*Tg(elavl3:H2B-GCaMP6s*)) we achieved co-expression of both the HaloTag protein and the calcium indicator. We then performed a two-step incubation using JF_552_ and JF_669_ to label neurons based on their birthdate (either before 2 dpf or between 2 and 5 dpf) and acquired the resulting two-color 2P images at 5 dpf (Fig. 4a–i, top and Supplementary Fig. 6a). To separate signals from the two fluorophores, we calculated the ratio between the two emissions and applied a demixing algorithm to subtract any potential leak from one dye into the opposite detection channel of the 2P microscope (corresponding to the two PMT detectors; see Material and Methods for more details).

Of note, the unique 2P excitation spectrum of JF dyes, characterized by a double peak with minimal absorption at ~920 nm^27^ makes them ideal for pairing with GFP-based calcium indicators. We confirmed this feature by assessing that the presence of the dye did not interfere with the detection of spontaneous calcium transients. Typically, the dye leakage into the PMT used for GCaMP detection was below 5%, indicating a negligible contribution to functional recordings (JF_525_: 27.5%, JF_552_: 0%, JF_585_: 4.7%, JF_669_: 0.21%; see Supplementary Fig. 5).

Next, to assess functional activity of neurons based on their birthdate, head-fixed larvae were presented with a broad range of visual stimuli while performing 2P calcium imaging of the GCaMP emission in the contralateral hemisphere of the OT (see Material and Methods). Calcium responses were first extracted and averaged over macro-regions containing the majority of early- or late-born Periventricular neurons (PVNs), the main cell type in the larval OT, whose somas are in the deepest layer of the tectum. Maturation state of the neurons was inferred based on the dye ratio (JF_552_/(JF_552_+JF_669_), Fig. 4a–i, see Material and Methods). We observed distinct differences in visually-evoked calcium responses between early- and lateborn neurons (Fig. 4a–ii), with stronger responses from earlyborn (more mature) neurons compared to lateborn ones, under most stimuli conditions (Fig. 4a–iii). Specifically, when exposed to a bright flash, both age groups exhibited similar fluorescence transients, suggesting comparable baseline visual sensitivity. However, the presentation of moving gratings elicited significantly greater responses in the earlyborn population relative to their lateborn counterparts (*P* < 0.05). Likewise, a looming stimulus triggered enhanced activity in the earlyborn neurons compared to the newborn cohort (*P* < 0.01).

Similar behaviors were observed at the single-cell level, using a broader and more complete set of visual stimuli, including dark and bright flashes, moving gratings and looming stimuli (see Material and Methods). After extracting the birthdate identity of individual neurons (JF_552_/(JF_552_/JF_669_); Fig. 4b–i), which matches the topographic organization shown in Fig. 1, their corresponding calcium traces were ordered according to dye ratios (Fig. 4b–ii, N = 5 fish). We then defined two subgroups from the full population (Fig. 4b–iii, see Material and Methods) and compared their average calcium responses. This single-cell analysis confirmed the population-level trends (Fig. 4a), and in general, earlyborn neurons exhibited higher visually-evoked activity. The most pronounced differences between the two neuronal age-groups were observed under gratings and looming stimuli (red sections in Fig. 4b–iii, see Material and Methods). Overall, earlyborn neurons showed a higher likelihood of activation, as indicated by a greater fraction of responsive cells across all the stimuli (lateborn: 40%; intermediate: 50%; earlyborn: 70%. *P* < 0.05, 1-way ANOVA; Supplementary Fig. 6b).

We then investigated the relationship between neuronal maturation and direction selectivity, by analyzing PVN’s calcium responses to gratings moving in 8 different orientations (see Material and Methods). We revealed a correlation between the age of individual neurons, their topographic organization and their directional tuning properties. Specifically, earlyborn neurons exhibited stronger and more consistent direction selectivity, while lateborn neurons showed reduced selectivity to moving bars (Fig. 4b–iv; *P* < 0.01; and Supplementary Fig. 6c). These findings, in accordance with previous studies, suggest that direction selectivity to gratings stimuli emerges progressively during development and is tightly correlated to neuronal maturation^13,42,43^. These observations are further supported by a complementary experiment in which we tracked the visual responses of neuronal subpopulations born at defined developmental stages (Fig. 4d). To achieve this, we performed either single or dual dye labeling with incubations at dpf *j* and *j+1*. This strategy allowed to identify birthdate-specific subpopulations, in particular neurons born between *j* and *j+1* dpf, defined by cells stained with only one of the two dyes. By performing calcium imaging the days following the incubations (*i* dpf), we were able to track birthdate-defined populations across larval development and follow the maturation of their visual responses (Fig. 4d).

As highlighted above, PVNs exhibit a clear topographical organization that correlates with their time of birth. In contrast, in other regions of the central nervous system, a neuron’s relative spatial position does not necessarily reflect its developmental maturity. CHLOK strategy allows to establish this correlation in such cases. For instance, within the tectal neuropil, Superficial Interneurons (SINs) and Neuropil Interneurons (NINs) constitute a heterogeneous neuronal population involved in diverse functions, and their spatial position does not reliably correlates with their time of birth^44–46^. However, by employing our dual-labelling protocol at 3 and 5 dpf, in combination with functional imaging, we were able to subdivide SINs and NINs based on their birthdate and distinguish their visual response profile accordingly (Fig. 4c).

Overall, these experiments validated CHLOK labelling strategy as a powerful tool to investigate the functional integration of neurons in the developing OT, enabling to discriminate their activity based on birthdate and following the gradual maturation of visual responses.

### CHLOK enables longitudinal tracking of neuronal age groups during functional maturation of the hindbrain

**Figure 5:**
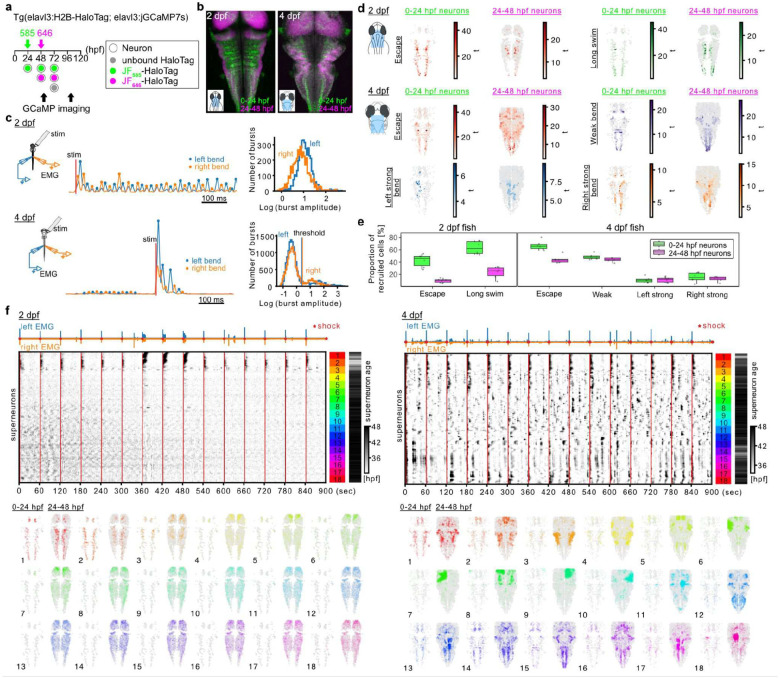
Visualization of functional maturation of neuronal age groups by CHLOK in the hindbrain **a,** Nuclei of neurons born before 24 hpf and those born from 24 to 48 hpf were visualized by Janelia Fluor 585 and Janelia Fluor 646, respectively. jGCaMP7s was expressed pan-neuronally. **b,** Visualization of the neuron group born before 24 hpf and that born from 24 to 48 hpf. **c,** Experimental setup for GCaMP imaging during fictive locomotion in 2 dpf and 4 dpf zebrafish. Electrical stimulus was delivered to the right-hand side of the head while monitoring muscle activity from both sides with EMG recording pipettes. Convoluted EMG signals from left (blue) and right (orange) axial muscles during a stimulus-evoked escape episode (2 dpf) and a spontaneous swim episode and a stimulus-evoked escape episode (4 dpf). EMG peaks corresponding to left and right bends were annotated with circles. Histogram showing the distribution of the EMG amplitude per bend for left (blue) and right (orange) bends. Vertical lines in the histogram for 4 dpf zebrafish indicate the thresholds to be considered “strong bend” in d. **d,** Longitudinal change of locomotor-related activity in each neuronal age group (top). Neuronal activity maps in 2 dpf zebrafish. Left: Neurons showing activity related to stimulus-evoked escape (Escape, red); Right: Neurons showing activity related to sustained swim observed in a portion of escape episodes (Long swim, green). Activation maps are separately generated based on the neuronal birthdate (Left, 0–24 hpf; Right, 24–48 hpf). Neurons below statistical significance (*P*_FDR_ = 0.05) are visualized in gray circles. Neurons above the significance are color-coded based on their t-values (bottom). Neuronal activity maps at 4 dpf. The panels are organized as in (i). Activation maps for stimulus-evoked escape (Escape, red), spontaneous swims with weak EMG bursts (Spontaneous swim, green), strong EMG bursts (Left strong bend, blue; Right strong bend, orange), and inter-swim intervals (Swim pause, purple). **e**, Percentage of neurons recruited during locomotor behaviors within each age group. Dots represent individual fish. Box and whisker plots were sorted based on developmental time points (2 dpf and 4 dpf fish) and neuronal age groups (0–24 hpf neurons and 24–48 hpf neurons). **f,** Emergence of functional classes in each age group (left). Functional classes in 2 dpf zebrafish. Top: Locomotor activity in response to repeated escape-inducing electrical shocks. Blue trace, Left EMG signal; Orange trace, inverted right EMG signal; Red circle, electrical stimulus. Electrical artifacts from the stimulus are removed (see Material and Methods). Middle: Neuronal activity sorted by Rastermap. Each row represents z-scored activity of superneurons. The average neuron age in each superneuron is represented in gray scale. Bottom: Maps of functional groups colored by position in Rastermap sorting. Each functional group is further divided into neurons born by 24 hpf and those born between 24 and 48 hpf. (right) Functional classes in 4 dpf zebrafish. The panels are organized as in 2dpf.

To link maturation of newborn neurons to behavioral development, we next focused on the hindbrain, which plays a critical role in the emergence of locomotor behavior^10^. We birthdated neurons born before 24 hpf and those born between 24 and 48 hpf, and assessed their activity using 2P Ca^2+^ imaging at 2 and 4 dpf (Fig. 5a, N = 6 fish per timepoint; see Material and Methods). The hindbrain structure changes significantly from 2 to 4 dpf, not just in size but also in the relative position of the two neuronal age cohorts (Fig. 5b), highlighting the importance of visualizing both age groups in individual fish. Consistent with previous reports^10,47^, we observed significant changes in the repertoire of locomotor behavior. Zebrafish at 2 dpf were capable of escaping in response to electrical stimuli but rarely swim spontaneously (Fig. 5c, 2 dpf, left; Fig. 5f, 2 dpf, top). Muscle activity also showed a unimodal distribution (Fig. 5c, 2 dpf, right), suggesting that zebrafish at this stage are limited in the range of muscle activation control. In contrast, 4 dpf larvae showed robust spontaneous swims besides escape responses evoked by electrical stimuli (Fig. 5c, 4 dpf, middle; Fig. 5f, 4 dpf, top). Muscle activity during the spontaneous swims was also distinctively weaker (Fig. 5c, 4 dpf, left), resulting in a broader range of muscle activity (Fig. 5c, 4 dpf, right). To assess how the activity of each neuronal age group changes during this behavioral development, we performed regression analysis to visualize correlated activity to locomotor events. In 2 dpf larvae, we looked for neurons that showed correlated activity to escapes or long swims, based on the variability of the length of escape episodes at this stage (Fig. 5f, 2 dpf, top), and found that both functional types are present in each neuronal age cohorts (Fig. 5d, 2 dpf). However, a much smaller proportion of the lateborn group showed activity related to these locomotor events (Fig. 5d, 2 dpf; Fig. 5e, 2 dpf, *P* < .0001, corrected for multiple comparisons, using a generalized linear mixed model for binomial distribution with subjects and locomotor types as random effects), suggesting that the lateborn group is still functionally immature. In 4 dpf zebrafish larvae, we looked for neurons that showed correlated activity not only to escape but also to locomotor events not present at 2 dpf, including spontaneous swim and strong lateralized response during spontaneous swim. As at 2 dpf, we found that each functional class exists in both neuronal age groups. However, a larger proportion of the lateborn group exhibited locomotor related activity (Fig. 5d, 4 dpf; Fig. 5e, 4 dpf, *P* < .0001, corrected for multiple comparisons), suggesting their ongoing functional maturation. To examine if this gradual maturation of the lateborn group also applies to other functional classes, we visualized the activity patterns across the two neuronal age groups at 2 and 4 dpf by sorting them based on their activity^48^. At 2 dpf, most prominent activity patterns were aligned with escape events and observed in the groups enriched in the earlyborn group (Fig. 5f, 2 dpf, Groups 1, 2). The rest of the neurons that did not show clear activity were mostly from the lateborn group (see the neuron age in Groups 3–18 in Fig. 5f, 2 dpf). At 4 dpf, a wider variety of activity patterns were observed, including the tectal neurons not captured in the regression analysis above (Fig. 5f, 4 dpf, Groups 5–10). These observations support the idea that the gradual maturation of the lateborn group applies generally across the examined brain regions. Interestingly, both analyses revealed that a non-negligible number of the earlyborn neurons were recruited in the activity patterns not present at 2 dpf (Fig 5d, 4 dpf; Fig. 5f, 4 dpf). This raises the possibility that coordination between neuronal age groups emerges during this period, which may play a critical role in the development of motor behavior. In conclusion, we showed that CHLOK enables longitudinal activity tracking of multiple neuronal age cohorts in a developing vertebrate and opened the avenue to investigating how their coordination contributes to behavioral maturation.

### Integrating CHLOK with sensors and actuators for birthdate-selective functional imaging and photoactivation

**Figure 6:**
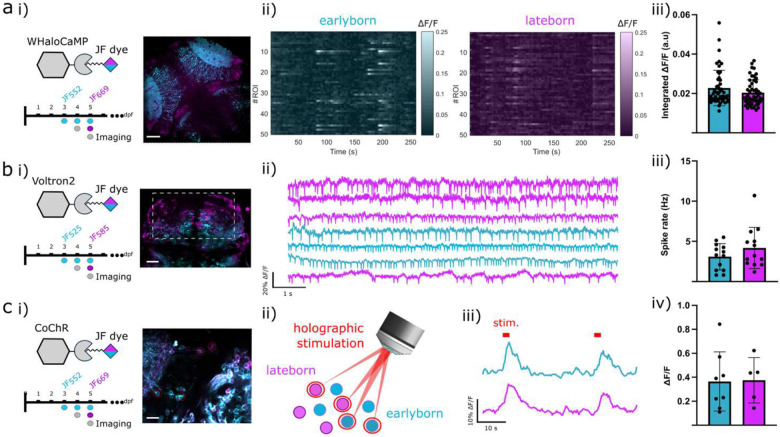
CHLOK-based birthdate selective tagging of optogenetics constructs. **a,** CHLOK applied to WHAloCaMP for birthdate-resolved calcium imaging. (i) Schematic of WHaloCaMP tagged by JF dyes and corresponding dual dye image of a WHaloCaMP-expressing larvae OT (JF_552_ incubated at 2 dpf, JF_669_ at 5 dpf; stable line). Scale bar: 50μm. (ii) Raster plot of ΔF/F traces from the two birth-selected populations acquired in sequential trials under dual laser spinning disk imaging. Recordings from the two populations (right) were performed sequentially with two different excitation wavelengths (561 nm and 642 nm, respectively). (iii) Barplot of the integrated calcium signal from individual traces of early- and lateborn neurons, cyan and magenta, respectively. Bar plot represent mean ± s.e.m (n = 50 cells per population; N = 2 fish). **b,** CHLOK applied to Voltron2 for birthdate-resolved voltage imaging. (i) Schematic of Voltron2 tagged by JF dyes and corresponding dual dye image of a Voltron2-expressing olfactory bulb (delimitated by white dotted rectangle; JF_525_ incubated at 2 dpf, JF_669_ at 5 dpf; transient expression). Scale bar: 25μm. (ii) Examples of Voltron2 fluorescence transients from the two birth-selected populations (early- and late-born) acquired in sequential trials under dual laser light sheet imaging. (iii) Barplots of the spiking rate from early and late-born neurons, cyan and magenta, respectively (mean ± s.d., n = 13 cells per population, N = 3 fish). **c,** CHLOK applied to CoChR opsin for birthdate-resolved optogenetic stimulation. (i) Schematic of CoChR opsin tagged by JF dyes and corresponding dual dye image of a CoChR expressing larvae (JF_552_ incubated at 2df, JF_669_ at 5 dpf). Scale bar: 20μm. (ii-iv) *In vivo* functional validation of birth-selective optogenetic stimulation in all-optical experiments on tectal neurons co-expressing CoChR-HaloTag and GCaMP6s (transient expression). (ii) After dual color birth-date based labeling (right schematic), earlyborn (cyan) and lateborn (magenta) neurons were selected for selective holographic photostimulation. (iii) Exemplary response traces from an earlyborn (cyan) and a lateborn neuron (magenta) showing calcium increases corresponding to photostimulation pulses (red ticks). (iv) Bar plot of the photostimulation induced ΔF/F peak amplitude from earlyborn and lateborn populations (n_552_ = 8 cells, n_669_ = 5 cells; N = 3 fish).

We next explored the versatility of CHLOK and its compatibility with various constructs, such as activity sensors and optogenetic actuators. In fact, a more practical application of CHLOK-based birthdate labeling could consist of directly color-code the fluorescence of an activity indicator. We first validated this strategy using WHaloCaMP, a chemigenetic calcium indicator in which the calcium-sensitive domain is fused with HaloTag^49^. We applied dual CHLOK labeling in WHaloCaMP-expressing larvae (JF_552_ at 3 dpf and JF_669_ at 5 dpf), followed by sequential functional imaging of each dye, enabling a selective monitoring of spontaneous activity in neurons with distinct birthdates (Fig. 6a). A similar strategy was used to achieve birthdate-selective tagging with the rhodopsin-based voltage indicator Voltron2, which uses a microbial rhodopsin as its voltage-sensing domain and Hatotag-JFdye as the reporter fluorophore^50^. Dual CHLOK labeling was applied to Voltron2-expressing larvae at 3 dpf (JF_525_) and 5 dpf (JF_585_), and fast fluorescent transients were acquired in the olfactory epithelium, a region known for reliable spontaneous spiking^50^. This enabled us to detect action potentials and extract firing rates in birth-resolved neuronal populations (Fig. 6b).

Taken together, these experiments highlight the power of CHLOK for birthdate-resolved calcium and voltage recording, and represent, to our knowledge, the first demonstration of voltage imaging in two age-defined neuronal subpopulations.

To further demonstrate CHLOK’s versatility, we extended it to optogenetic actuators for all-optical experiments, where the combination of opsins and activity indicators enables the simultaneous optical manipulation and readout of cellular activity in a non-invasive manner^51,52^. These experiments typically require careful separation of the excitation and emission spectra of opsins, their fluorescent tags and the activity indicators, particularly challenging when labeling multiple subpopulations and under 2P excitation, due to higher spectral overlaps. This can lead to unintended opsin activation during functional imaging or cell screening, and generally limits flexibility in combining constructs and in multi-cells targeting^51^. To address some of these limitations, we developed a novel design by fusing the opsin CoChR with HaloTag. We first evaluated the construct in CHO cells expressing CoChR-HaloTag, and recorded photocurrents during 2P photostimulation and simultaneous imaging at different wavelengths (Supplementary Fig. 7). The orthogonality between CoChR and the peculiar 2P absorption profile of JF dyes (here JF_585_; with peaks at 800 nm and >1000 nm) allowed for bright imaging with minimal artifactual photocurrents from the imaging laser. In contrast, the imaging of GFP-tagged opsins, requiring 950 nm excitation, induced significantly larger artifactual photocurrents (>200 pA), potentially leading to unwanted depolarization during cell screening. We next tested the *in vivo* functionality of this construct by co-expressing CoChR (with transient expression) with GCaMP6s, and performing simultaneous 2P imaging and 2P holographic photoactivation of user-defined sets of cells. To identify opsin-expressing neurons, we applied dual CHLOK labeling (Fig. 6c–i; JF_552_ at 3 dpf, and JF_669_ at 5 dpf), and due to spectral selectivity in both excitation and emission, HaloTag labeling could be readily separated. Using 2P holographic stimulation, we then selectively targeted either early- or late neurons (Fig. 6c–ii) while recording GCaMP calcium responses from the full field of view. Photo-induced calcium responses were clearly detected in targeted neurons (Fig. 6c–iii), confirming successful activation. The average fluorescence changes ΔF/F were comparable between the two populations (0.20 ± 0.02 for earlyborn and 0.22 ± 0.02 for lateborn neurons; Fig. 6c–iv), suggesting similar levels of opsin expression and comparable neuronal excitability.

This last set of experiments demonstrates the capability of CHLOK for all-optical manipulation of activity in birthdate-resolved subpopulations, opening new avenues for dissecting their functional incorporation into emerging neural circuits.

## Discussion

In this study, we developed CHLOK, a novel neuronal birthdating strategy designed to overcome longstanding challenges of studying the developmental trajectory of individual neurons and the maturation of their functional roles within neural circuits. By combining HaloTag technology with JF dyes, this approach enables, for the first time, the tracking of the entire population of neurons differentiating within a specific time window in a living animal.

We demonstrated that this strategy not only addresses key limitations of existing birthdating methods but also introduces a powerful platform for integrating birthdate labeling with a large variety of investigations, including subcellular fluorescent imaging, simultaneous visualization and functional interrogation of multiple age cohorts, longitudinal activity tracking and combination with sensors and optogenetics tools.

The most widely used birthdating approach relies on DNA synthesis markers such as BrdU and EdU, which label all actively dividing cells. However, the visualization of these markers requires tissue fixation, making it incompatible with *in vivo* studies. Furthermore, the markers are inherited to all sibling cells that emerged from multiple rounds of cell division, unlike CHLOK that specifically targets differentiating neurons. This difference is likely the cause of the more distinct birthdate-specific patterns observed with CHLOK. Other existing *in vivo* birthdating methods, such as timed genetic labeling of progenitor cells by electroporation^53,54^ or heat shock^55,56^, face similar issues of labeling all the sibling cells, necessitating sparse labeling for interpretability. More sophisticated approaches have been developed to sequentially switch reporters in synchrony with the cell division cycle^21,22^, but the yield of lateborn cells tagging is limited due to their complexity. In either case, the labeling is stochastic by nature and incomprehensive. In contrast, CHLOK offers consistent and comprehensive labeling, providing a reliable and precise tracking of the chronological organization of full neuronal populations at single cell-resolution*, in vivo*.

These features, combined with the simplicity and amenability of HaloTag-based engineering, opens a wide range of new applications and seamless integration with a growing suite of molecular tools.

As a first example, we demonstrated a unique subcellular visualization of neuronal age groups, revealing distinct chronological organizations of axonal and dendritic processes across the vertebrate brain. This approach will enable to visualize the anatomical maturation of emerging neurons, especially when paired with cell-type specific targeting.

We further extended CHLOK to functional imaging by coupling it with HaloTag-based calcium and voltage indicators, enabling direct functional readout of neurons based on their birthdate. While optimization is needed to enhance sensitivity and dye compatibility, this proof-of-concept establishes a new paradigm for functional analysis of neuronal age cohorts. Yet another example is HaloTag-based cellular manipulation, for which we directly labeled opsins with CHLOK and achieved the first targeted 2P photostimulation of birthdate-defined neuronal subsets. Combined with recently developed HaloTag-based photoablation, similar protocols may also enable to ablate age-specific cellular population^57^. Taken together, CHLOK offers a straightforward way to combine reliable and precise birthdating with the expanding repertoire of HaloTag-based tools.

We took advantage of the wide range of biocompatible JF dyes to demonstrate simultaneous visualization of up to five neuronal age cohorts within the same animal. This level of multiplexing opens powerful new avenues for investigating how genetic and epigenetic differences across individuals contribute to the dynamics of neurogenesis and circuit integration. Furthermore, the different JF dye’s spectral properties ensure compatibility with a variety of fluorescent proteins and optogenetic constructs. Since the HaloTag protein itself is non-fluorescent, this allow the generation of transgenic lines that are fluorescence-neutral, allowing the user to choose the most suitable dye based on the fluorescent protein or opsin required for the experiment. In particular, the minimal absorption of JF dyes at 920–950 nm makes CHLOK highly suitable for combination with the most widely used GFP-based Ca^2+^ indicators and emerging GFP-based voltage indicators^58,59^.

We indeed examined the developmental trajectory of multiple age cohorts at activity level in the OT and the hindbrain, combining CHLOK multilabeling with 2P GCaMP activity recording. We showed that newborn neurons in the OT, irrespective of their birthdate, exhibited increasingly visual responses as they matured and became functionally similar to the earlyborn, pre-existing neurons. Newborn hindbrain neurons also became functionally mature in a similar time course but exhibited activity distinct from the pre-existing neurons, which also change their activity as they mature, suggesting potential interactions across age groups. In sum, both brain regions showed a similar rate of maturation of newborn neurons yet exhibited distinct functional roles of newborn neurons. Similar functional investigation across brain regions will be required to understand the roles and mechanisms of the distinct maturation process.

CHLOK also proves to be compatible across a wide range of temporal scales and has the potential to be employed for animal models other than zebrafish. Our time-resolved labeling protocol, with a 2-hour pulse dye incubation separated by 12 hours to a few days, was well-suited to the rapid neurogenesis and circuit development observed in zebrafish larvae. However, thanks to the prolonged persistence of the labeling (over weeks), the same approach could be adapted for slower-developing models by matching the incubation timeline accordingly.

Although we focused on zebrafish to demonstrate CHLOK’s capabilities due to the high bioavailability and efficient skin penetration of the dye, the mechanism behind CHLOK applies to other animal models. Indeed, a similar dye loading can be used *in vivo*, for instance, in C. elegans^60^, flies^61^, and mouse^50^. With the ongoing development of more bioavailable JF dyes^57,62,63^, multiplexed CHLOK is increasingly feasible.

In conclusion, our novel birthdate labeling strategy CHLOK, based on HaloTag and bright JF dyes, provides a powerful, flexible, and non-invasive tool for studying *in vivo* neuronal integration, functional activity, and circuit development in the vertebrate brain, potentially extendable across multiple model organisms. This approach offers a significant advance over traditional methods by enabling single-cell resolution birthdate mapping, functional imaging, and all-optical control, opening new avenues for the investigation of brain development and plasticity.

## Material and Methods

### Animals

All procedures involving zebrafish were conducted in accordance with national and European (2010/63/EU) guidelines, the animal research guidelines from the Canadian Council on Animal Care and the University of Toronto Local Animal Care Committee. They were approved by the authors’ institutional review boards and national authorities (French Ministry of Research, protocol ID: APAFIS#21323; University of Toronto, protocol ID: 20012777). Zebrafish were obtained from an in-house breeding colony of wild-type, transgenic, and mutant adults maintained at 28.5 °C on a 14 h light/10 h dark cycle with sunrise and sunset. Embryos were raised in a separate incubator but at the same temperature and on the same light-dark cycle (in fish water: 0.3 g/L Instant Ocean). Fertilized embryos were collected every hour and housed in separate Petri dishes to determine the time of fertilization at the temporal resolution of hours. The housing density was maintained lower than 60 embryos per 10 cm petri dish to ensure optimal growth. Embryos were staged^64^ and only the ones with normal development were used in the study. Juvenile fish were kept in the fish facility until the day of the experiment (transferred in becher with fish water and kept in the incubator before imaging).

### Plasmid and transgenic lines generation

We generated different Halotag-based constructs by subcloning (Gibson Assembly Cloning Kit) Halotag and other specific sequences and inserting them into various promoters: *p5’ QUAS*^65^; *Xla.Tubb2b-hsp70-ubc*, *elavl3* and *14UAS*. Halotag was subcloned alone or with H2B sequence, for nuclear expression. In some cases, we used, as reporters, the zebrafish *hatching enzyme 1, tandem duplicate 1 (he1.1)* promoter^65^, driving CFP or mCherry expression in the hatching gland, or the *myosin light chain (myl7)* promoter^66^, driving the expression of GFP in the heart. The following previously published transgenic lines were used: *Tg(xla.Tubb2b:QF2)*^65^, *Tg(elavl3:H2B-GCaMP6f)*^67^; *TgBAC(slc17a6:EGFP)*^68^, hereafter refer to as *Tg(vglut2a:EGFP)*); *Tg(elavl3:Voltron-ST)*^69^, the last two of which were propagated to the nacre background. The transgenic lines under *14UAS* promoter were generated using the Tol1 transposon system, while the ones under the *Xla.Tubb2-hsp70-ubc* promoter were generated using the Tol2 transposon system. The following transgenic lines were generated using the Tol2 transposon system: *Tg(elavl3:H2B-HaloTag)*, *Tg(elavl3:msyp-HaloTag)*, with plasmids generated by cloning the corresponding reporters to pTol2-elavl3:Voltron-ST^69^. H2B-HaloTag and msyp-HaloTag were subcloned from piggybac-EF1-Halo-H2B and piggybac-hsyn-msyp-Halo-neo, respectively (both of which are generous gifts from Dr. James Zhe Liu). *Tg(elavl3:H2B-HaloTag)* and *Tg(elavl3:jGCaMP7s)* were propagated to the cancb1 ts25/+ mutant background^70^ and maintained as heterozygous mutants. The WhaloCaMP plasmid was taken from^49^ and the Voltron2 sequence from^50^ (generous gift from Dr Takashi Kawashima) and subcloned in the QUAS promoter expressing *he.1.1:CFP*.

Experiments were performed on stable transgenic lines or in embryos transiently expressing the plasmids, as specified in the main text or figure legends. The DNA constructs(25 ng/uL) were co-injected with tol1 (for 14UAS promoter) or tol2 (for Xla.Tubb2:QF2-hsp70-ubc and elavl3 promoters) transposase mRNA (25 ng/uL) in one-cell stage of Casper^71^ mutants zebrafish embryos. Founders were identified by screening progeny for fluorescent hatching gland, bleeding heart cells or JF dye fluorescence directly.

The different generated plasmids and stable lines are listed in Supplementary table 1:

### JF dye incubation and preparation of zebrafish larvae

To label Halotag-expressing neurons the embryos were incubated in a solution containing the fluorescent Halotag ligand JF dye [3.3 μM JF dye and 0.033 % DMSO; unless differently specified, see below] in fish rearing water at room temperature for two hours. The incubation was performed at different moments of larva maturation (indicated in terms of hours or days post fertilization, hpf or dpf). After screening for the fluorescence of the JF dye in the brain, the fish were returned to fish rearing water in the incubator until the time of the experiment.

### Comparison of HaloTag birthdating and EdU labeling

#### Single-pulse HaloTag birthdating

Two JF dyes were loaded in 24-hours separation in the transgenic line expressing HaloTag as well as GCaMP6f in the nuclei of differentiating neurons with the *elavl3* promoter to distinguish neurons born before the first dye loading from those born between the first and second dye loading. JF_635_ was chosen as the second dye due to its fluorogenic property. This allows us to minimize the signal from unbound JF dye molecules when we image fish right after the second dye loading. JF_585_ was chosen as the first dye based on its brightness and clear separation from the fluorescence signals from GCaMP6f and JF_635_. The dye loading procedure is based on^26^ with a few modifications. The 0.3 μM dye solution was prepared by dissolving 1.67 μL of 1 mM DMSO stock solution in 5 mL of system water. Fish were incubated in the 0.3 μM solution for two hours and then rinsed thoroughly with system water before transferring them to a new 10 cm culture dish filled with clean system water. Embryos before hatching were dechorionated to facilitate the washout of the unbound dye molecules from embryos. Five neuronal age groups were targeted in this experiment: Group 1, neurons born before 24 hours post-fertilization (hpf); Group 2, neurons born from 24 to 48 hpf; Group 3, neurons born from 48 to 72 hpf; Group 4, neurons born from 72 to 96 hpf; Group 5, neurons born from 96 to 120 hpf. All groups were imaged between 122 and 126 hpf using a confocal microscope (Zeiss, 710/800). Fish were anesthetized in tricaine methanesulfonate (MilliporeSigma, E10521) dissolved in system water at 160 mg/L (hereafter referred to as MS-222) and then embedded in 1.6% low-melting-point (LMP) agar (MilliporeSigma, 2070-OP). Volumetric images of the rostral and caudal halves of the brain were imaged separately with a 20x 1.0 NA objective lens (Zeiss, W Plan-Apochromat 20x/1.0) and then stitched together with the stitching plugin available through Fiji^72^.

#### EdU labeling

EdU labeling was performed using Click-iT EdU Alexa Fluor 647 Imaging Kit (ThermoFisher, C10340) with modifications. Fertilized eggs from *Tg(elavl3:H2B-GCaMP6f)* were mounted based on developmental stages. Embryos at 4 hpf were placed on the standard egg injection mold used for single-cell DNA injection. Embryos at 24 hpf were dechorionated and embedded in 0.6% LMP agar. Zebrafish at 48 hpf and onward were embedded in 1.2% LMP agar. Then 0.8 nL bolus of 10 mM EdU solution was pressure injected into the yolk via a glass micropipette connected to a microinjector (Eppendorf, FemtoJet). Assuming all the EdU molecules were retained in the body, this translates to less than 5 ng/ul of EdU concentration in the body, which is less than the bath concentration of thymidine analogues used in past zebrafish studies^73^. Zebrafish were transferred back to a new culture dish filled with clean system water and kept in an incubator until 120 hpf when they were sacrificed by an overdose of 0.04% MS-222, then immediately fixed overnight in 4% PFA in 0.1 M phosphate buffer (pH 7.4) at 4 °C. Following 1xPBS rinses, the brain was exposed with a fine tungsten pin as described previously^74^. The subsequent staining procedure follows the procedure described in the kit, except the fish were kept in the cocktail without the buffer additive at −20 °C for an hour before starting the reaction with the buffer additive at room temperature. This additional step minimized the signal amplification biased toward the brain’s surface. The stained brains were imaged under a custom two-photon microscope described previously^10^ to obtain homogeneous signal across the entire depth of the brain for both GCaMP and Alexa Fluor 647, which were excited with a femtosecond laser tuned to 830 nm (MKS Instruments, Mai Tai HP, Deep See, MA). The entire brain was imaged at once using a 16x objective lens (Nikon Instruments, CFI75 LWD 16x W, NY).

### Single-pulse HaloTag birthdating of subcellular structures

To visualize presynaptic terminals of neuronal age groups, we employed *Tg(elavl3:msyp*-*HaloTag)*, the elavl3 transgenic line expressing HaloTag with synaptophysin localization sequence^37,38^. To emphasize the signal from dendritic processes, we chose the elavl3 transgenic line expressing HaloTag-based voltage indicator with Kv2.1 localization sequence since this sequence have been shown to target the tagged protein to somatodendritic processes^39–41^. They were subjected to the same single-pulse labeling procedure based on two JF dyes, JF_585_ and JF_635_, as described above and the data were processed similarly. To ensure precise registration of subcellular structures, these transgenic lines were crossed with *TgBAC(vglut2a:GFP)* that exhibit GFP signals broadly in the neuropil area. The whole-brain volumes were acquired using the same imaging and stitching protocol as described *in Single-pulse HaloTag birthdating*.

### Multi-pulse HaloTag birthdating

To visualize all five age groups examined above in the same animal, a series of JF dyes was loaded to the developing *Tg(elavl3:H2B-HaloTag)* in 24-hours intervals: Janelia Fluor 525 (JF_525_) at 24 hpf, JF_585_ at 48 hpf, Janelia Fluor 669 (JF_669_) at 72 hpf, Janelia Fluor 549 (JF_549_) at 96 hpf, and JF_635_ at 120 hpf. The dye loading protocol is identical to the one described above. However, based on the observation that neuronal age groups at similar age are also close in space, the order of the dyes was chosen to maintain reasonable separation of excitation and emission spectrum across neighboring age groups. We also observed in preliminary experiments that the loading of JF_525_ at a later developmental time point resulted in the labeling of neurons near the blood vessels (data not shown), leading us to use it as the first dye. JF_635_ was chosen as the last dye due to its fluorogenic property as described above. The imaging procedure is also similar to the one described above except that we used an inverted confocal microscope equipped with white-light laser (Leica, Steralis, Germany) to excite each of the five dyes at the optimal wavelength. Fish were mounted in 1.6% LMP agar ventral-side up and imaged with a 25x 0.95 NA objective lens (Leica, HC FLUOTAR L 25x/0.95 W VISIR, Germany).

### Optical systems

#### Confocal microscope

Five-dye multi-pulse birthdating images in Fig. 2 were acquired with a Leica Steralis system equipped with a white-light laser. JF_525_, JF_549_, JF_585_, JF_635_, and JF_669_ were excited at 525, 549, 585, 635, and 669 nm, respectively. Fluorescent images reported in Fig. 3 and Supplementary Fig. 1,4 were acquired using a commercial confocal microscope (Olympus FV3000). Multiple laser sources (488, 514, 561, 594, 640 nm) and customizable wavelength detection windows (based on spatial dispersion of the signal and a multidetector array) were available, allowing for the selective excitation and detection of individual JF dyes. Two-dye single-pulse birthdating images presented in Fig. 1 and 2 were acquired using Zeiss 710 or 800. GCaMP/GPF, JF_585_ and JF_635_ were excited with 488, 561, and 640 nm lasers, respectively.

#### Two-Photon Imaging

Two-photon imaging was performed using two different 2P scanning microscopes. For Fig. 4, we used a commercial model (LAVision, Miltenyi Biotec) combined with a tunable Ti:Sapphire femtosecond laser (Coherent Chameleon, wavelength range 700–1100 nm) and visual stimulation. JF dye imaging was typically performed at 800–850 nm, using dedicated band-pass filters in front of the PMTs (typically a 525/50 for the green PMT, 595/40 or 700/40 (exchangeable) for the red PMT) to minimize crosstalk in the presence of multiple dyes and to avoid contamination of the GCaMP recordings. Calcium transients were acquired using galvo-galvo bidirectional 2P scanning at 3 Hz at 920 nm with a FOV of 300×300 μm. A slightly different configuration was used for imaging EdU-labeled fixed samples and functional recordings during locomotion behavior (Figure 4), consisting of a custom microscope equipped with a resonant scanner (Thorlabs, MPM-2PKIT), a piezo objective scanner 267 (PI, P-725K129, Germany) and a 16x objective lens (Nikon Instruments, CFI75 LWD 16x W, NY) controlled by ScanImage (Vidrio Technologies, VA). For the imaging of EdU-labeled samples, GCaMP6f and Alexa Fluor 647 were excited with a femtosecond laser tuned to 830 nm (MKS Instruments, Mai Tai HP, Deep See). During the functional imaging, all the fluorophores were excited with a femtosecond laser tuned to 940nm (MKS Instruments, Mai Tai HP, Deep See, MA) to prioritize the functional GCaMP signal. Multi-channel functional volumes (512 × 256, 30 slices, 7 mm z step) were acquired for a period of 30 min at a volume rate of 2 Hz. Volumetric Ca^2+^ imaging of the caudal half of the brain was performed at 48–56 hpf (referred to as 48 hpf) and 96–104 hpf (referred to as 96 hpf) similarly to^10^.

#### One-Photon Spinning Disk imaging

Calcium imaging activity in WhaloCaMP-expressing larvae (Fig. 6a) was performed using a confocal spinning microscope on an upright microscope (Gataca W1). JF_552_ and JF_669_ were imaged with respectively a 561nm and a 642 nm laser. Emission sets filters used were EX BP 482/35, BS FT 506, EM BP 536/40 and EX BP 543/22, BS FT 562, EM BP 593/40, respectively. Spinning disk time series were acquired at 5 Hz with a FOV of 330 × 330 μm and using a X40 objective (NA = 1.15).

#### Light-sheet for voltage imaging

Voltage measurements were performed using a custom light-sheet imaging system (Fig. 6b). Single-plane illumination was generated by an elongated, low-NA beam focused by an underfilled EC Plan-Neofluar 5x/0.16 Zeiss objective, and horizontally scanned using a galvo mirror. The detection path included an XLUMPLFLN 20x objective (Olympus) and a Kinetix camera (Teledyne). Multiple excitation wavelengths were provided by a fiber-coupled laser combiner (L6Cc-CSB-1311-0-488-522-561-638-0-400, Oxxius, France). Voltron_525_ was excited at 488 nm and detected through a 562/40 filter, while Voltron_585_ was excited with a 561 nm laser and detected using a 593 lp filter. All measurements were acquired for 10 seconds at 500 Hz, over a field of view of approximately 100 × 300 μm, centered on the olfactory epithelium.

#### All-optical experiments using holographic photostimulation

The experiments of Fig. 6c were performed using a custom-built microscope in a configuration similar to that used for all-optical recording in^75^. Briefly, the microscope enabled simultaneous 2P scanning imaging (using a Ti:Sapphire infrared laser, Mai Tai, Spectra Physics) and holographic photostimulation (using a fiber amplifier laser at 1030 nm with a 500 kHz repetition rate, Satsuma, Amplitude Systems). Holographic light shaping, based on the use of a spatial light modulator (SLM), allowed for the reshaping of the 2P stimulation laser to target specific regions of interest. In this setup, the system was capable of generating multiple diaphragmed Gaussian spots with an almost top-hat intensity profile, approximately 10μm in diameter. Combined with temporal focusing^76^, this configuration maintained good axial confinement of the excitation (~15μm), enabling near single-soma resolution. The setup supported both conventional static illumination and a more efficient, cyclic illumination approach known as FLiT^75^. Based on an initial fluorescence image, earlyborn and lateborn neurons expressing the CoChR opsin were identified. While performing 2P calcium imaging, pulses of holographic illuminating (10 pulses, 30 ms, 10 Hz) were delivered selectively to the chosen neurons. Typically, 1 to 10 neurons per FOV were targeted. Photostimulation powers per cell range between 5 to 20 mW (power under static illumination).

### Visual Stimulation

Zebrafish larvae were paralyzed by 3–5 minutes incubation in α-bungarotoxin (1 mg/mL) and mounted in a 35mm petri dish using 2% (v/v) low melting point agarose, and maintained at room temperature during all the recording duration. Visual stimuli were delivered using a red-emitting projector, shining on a diffusive screen located at a distance of ~3 cm from the larva’s head. Simultaneously, the corresponding projected pattern covered a 120° horizontal visual field. Slightly different stimulus parameters were performed in experiments reported in Fig. 4. For Fig. 4a, the sequence of visual stimuli was: (1) Bright Flash: a single 1-second bright flash stimulus; (2) Bright Ramp: a gradual increase in brightness over 5 seconds; (3) Gratings: four gratings corresponding to the cardinal directions (0°, 90°, 180°, and 270°) with 10°-wide dark stripes moving at 20°/s. Each grating was presented statically in the first set of stimuli, then in motion for 5 seconds in the second set of stimuli); (4) Looming Stimuli: a steady looming, a dark circular dot whose dimension expands at a constant rate of 20°/s, followed by an exponential looming, with lateral dimensions doubling every second); (5) Dark Ramp: a gradual decrease in brightness over 5 seconds. For Fig. 4b, the sequence of visual stimuli was: (1) Dark Flash: four 1-second dark flashes with increasing contrast, spaced 20 seconds apart, followed by a transition to a dark background lasting 20 seconds; (2) Bright Flash: four 1-second bright flashes with increasing contrast, spaced 20 seconds apart, followed by a transition to a bright background; (3) Gratings: eight gratings presented in different orientations (0°, 45°, 90°, 145°, 180°, 225°, 270°, 315°. 0° = caudal to rostral and 90° = ventral to dorsal), each with 10°-wide dark stripes moving at 20°/s. Gratings were static for 20 seconds before moving for 5 seconds; (4) Looming Stimulus (an exponential growth pattern with a radius doubling time of 1 second). Stimuli were programmed and synchronized with calcium imaging recordings, ensuring precise timing and reproducibility.

### Longitudinal functional imaging of locomotor activity in birthdated hindbrain neurons

*Tg(elavl3:H2B-HaloTag)*^*ts25/+*^ and *Tg(elavl3:jGCaMP7s)*^*ts25/+*^ were crossed to obtain *Tg(elavl3:H2B-HaloTag;elavl3:jGCaMP7s)*^*ts25/ts25*^ to perform electromyogram (EMG) recording in a paralytic fish. Fish with the paralytic phenotype were dechorionated at 24 hpf and separated for subsequent Halotag birthdating and functional imaging. The embryos were loaded with JF_585_ at 24 hpf and JF_646_ at 48 hpf using the procedure described in single-pulse birthdating. We used JF_646_ as the second dye since preliminary experiments found it challenging to get enough fluorescence from JF_635_ with our two-photon microscope. Volumetric Ca^2+^ imaging of the caudal half of the brain was performed at 48–56 hpf (referred to as 48 hpf) and 96–104 hpf (referred to as 96 hpf) as in^10^ with a few modifications. Fish were mounted in 1.6% LMP agar dorsal side up, with the tail and right side of the head freed from agar. A concentric platinum iridium stimulation electrode (Microprobes, PI2CEA10–200, MD) was placed on the right side of the head to elicit escape towards the left with a brief electrical stimulus. A glass micropipette was placed on each side of axial muscles near anus to record alternating EMG activity during locomotion. The size of the micropipette tip was around 20 μm to minimize the contamination of the EMG signal from the other side, which was noticeable when fish exhibited a strong EMG signal during escape. The signal was amplified (100x) and band-pass filtered (100–1000 Hz) through an extracellular amplifier (NPI, EXT-02B, Germany) and digitized at 6 kHz (National Instruments, PCIe6363, TX). A brief electrical stimulus (0.1 ms in duration, 2–4 V, 1 min interval) was delivered through a glass electrode placed on the side of the head using a stimulus isolator (Digitimer Ltd., DS-2A, England). The stimulus amplitude was adjusted to evoke strong motor activity consistently before the start of the functional imaging. After the functional imaging, a high-quality structural volume was acquired using a femtosecond laser tuned to 1120 nm (MKS Instruments, Insight, Deep See, MA) to excite JF_585_ and JF_646_ more optimally alongside GCaMP excited at 940nm.

### Preparation of CHO cells and patch clamp recordings

CHO cells were acquired from Sigma (Sigma, 85050302) and cultured in T25 flasks (Falcon, 353107) in a medium consisting of DMEM-F12 + Glutamax (Fisher, Gibco^™^ 10565018), supplemented with 10% SBF (Fisher, Gibco^™^ 10500064) and 1% penicillin/streptomycin (5000 U ml−1). Cells were passed every 2–3 days, until P20 to avoid genetic drift. Prior to each experiment, cells were seeded on coverslips (Fisher, 10252961) in 24-well plates (25,000 cells/ml). After 24 hours, cells were transiently transfected with the plasmids pAAV-SynCoChR-HaloTag or pAAV-hSyn-CoChR-GFP. The medium was then replaced after 4 hours. Experiments reported in Supplementary Fig. 7 were performed 48–72 hours post transfection. 1-hour prior to electrophysiological recordings, CHO cells expressing the CoChR-HaloTag were stained with the fluorescent dye JF_585_ (200nM). Whole-cell voltage-clamp recordings of opsin-positive cells (GFP-positive or JF_585_-positive) were performed at room temperature (21−23 °C) on the home-made holographic microscope described above. Cells were visualized using infrared differential interference contrast (IR-DIC) and camera imaging. Cells were continuously perfused with artificial cerebrospinal fluid (ACSF) comprised of 125 mM NaCl, 2.5 mM KCl, 1.5 mM CaCl2, 1 mM MgCl2, 26 mM NaHCO3, 0.3 mM ascorbic acid, 25 mM D-glucose, 1.25 mM NaH2PO4. Continuous aeration of the recording solution with 95% O2 and 5% CO2, resulted in a final pH of 7.4. Recording pipettes were made from borosilicate glass capillaries using a horizontal puller, resulting in a resistance of 3–5 MΩ when filled with the internal solution, consisting of 135 mM K-gluconate, 4 mM KCl, 4 mM Mg-ATP, 0.3 mM Na2-GTP, 10 mM Na2-phosphocreatine, and 10 mM HEPES (pH 7.35). Recordings were obtained using a Multiclamp 700B amplifier, filtered at 2 kHz, and digitized at 10 kHz with a Digidata 1440A interface (Molecular Devices). Data acquisition and analysis were performed using pClamp software (Molecular Devices). Scanning 2P imaging was performed at full field (FOV ~350 μm) with a bidirectional scanning at 3 Hz.

### Imaging on fixed samples from juveniles’ tissues

Larvae or juvenile fish were incubated in JF dyes (1 or multiple dyes) at specific time points as indicated in the manuscript. At specific time points, juvenile fish were prepared for fixation. To do so, they were euthanized using an overdose of 0.2% Tricaine methanesulfonate (MS-222, concentration ≥ 250 mg/L) and ice-cold fish water and the head was gently cut, fixed in paraformaldehyde 4% in PBS overnight at 4°C and then washed three times for 10 min in PBS containing 0.1% Triton X-100 (PBST). Subsequently, the brain was carefully dissected under a stereomicroscope (by taking care of removing any tissue adhered to the brain) and transferred into a solution of 30% sucrose overnight at 4 °C, for cryoprotection. The next day, they were transferred to plastic molds and embedded in O.C.T compound (Optimal Cutting Temperature; embedding medium for cryotome; Sakura Finetech) after removal of the sucrose. Blocks were then frozen on dry ice and stored at −80°C. Samples (brains or retinas) were cut with a cryostat at 12–16μm thickness in the coronal plane. The sections were mounted on Fisherbrand Superfrost Plus slides (Fisher Scientific) and kept at 4°C before confocal imaging.

### Data Analysis

#### Static Fluorescent Images

For protocols involving incubation with multiple dyes in the same larvae, efforts were made to minimize crosstalk in both excitation and detection of different dyes. Using a confocal microscope, we fine-tuned excitation and detection settings for each dye, achieving negligible crosstalk among the typical JF_525_, JF5_552_, and JF_669_ dye combination.

In 2-photon imaging, the overlap in excitation spectra (with dyes excited at 800–850 nm) and the use of only two PMTs resulted in some fluorescence crosstalk. To address this, the fluorescence intensities of individual dyes were measured in each PMT (with different emission filters) using larvae expressing single dyes. This allowed us to estimate the detection efficiency of each fluorophore and assess potential crosstalk across detection channels. These intensity values were then used as coefficients in a linear wavelength-demixing algorithm, enabling the isolation of the signal contributions and intensities of each individual dye.

#### HaloTag Birthdating Analysis

Both HaloTag birthdating and EdU labeling datasets were registered to the *Zebrafish Brain Browser* (ZBB) atlas^36^ using *Greedy* registration program (https://github.com/pyushkevich). Each channel from an individual 3D volume was converted to a NIfTI file format. Then the channel corresponding to *Tg(elavl3:H2B-GCaMP6f)* or *Tg(vglut2a:EGFP)* was used as a source to register all the channels to the ZBB atlas using huc-h2b-rfp-ref-01.nii.gz or vglut-ref-01.nii.gz available from *zenodo* as a reference volume. The detailed sequence of Greedy commands is shown below (Listing 1). The -sv option was enabled to obtain better behaved deformation fields (https://github.com/pyushkevich).


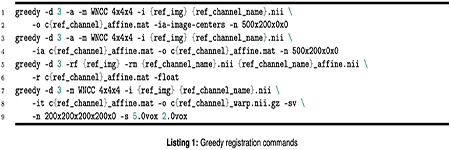


When the registration failed before warping, we edited the header of the source NIfTI files to bring them close to the reference volume using SPM12 (SPM12 Software - Statistical Parametric Mapping; https://www.fil.ion.ucl.ac.uk/spm/software/spm12/). The registered source file was compared to the reference file. When the boundaries between inside and outside the brain or between the cell body and neuropil area did not match, lower smoothing kernel (-s) parameters were set to increase the flexibility of the warping. When the cell bodies are excessively elongated, higher smoothing kernel parameters were set. The stacks that were not registered properly were excluded from the subsequent analysis.

For the HaloTag birthdating data, each channel file was normalized between 0 and 1 individually based on the median voxel value which corresponds to the background signal and the top 0.01 percentile voxel value (the values below 0 were set to 0 and the values above 1 were left untouched). After this normalization, these channel files were brought to the ZBB atlas space by applying the affine transformation and warping determined by Greedy. Then the neuronal age group of interest was visualized by subtracting the normalized channel of the preceding dye from the normalized channel of the dye of the interest. The values below zero were set to zero. The population image for each age group was generated by calculating the median of the subtracted images across all individual fish in the group (N ≧ 4 fish in all conditions). In the case of subcellular HaloTag labeling, these processing steps highlight the neuropil area exclusively occupied by the neuronal age group of interest, due to the across-subject variability of individual neurons and the resolution limit of optical microscopy.

For the multi-pulse HaloTag birthdating, the acquired volumes were fed to the same registration and processing steps used for single-pulse HaloTag birthdating for consistency. A minor modification was made to generate the virtual first dye signal for each target group: the ‘first’ dye signal was defined as the maximum of the normalized channels corresponding to the previous age groups and subtracted from the channel corresponding to the target group.

For the EdU staining data, the channel file corresponding to EdU was normalized as described above and the median of the normalized EdU signal across individual fish was used as the population data (N ≧ 4 fish in all conditions).

#### Calcium Imaging Analysis during visual stimulation

Calcium transients from GCaMP fluorescence signals were analyzed either by integrating over macro-regions or by extracting signals from individual cell bodies: (i) Macro-regions were defined as areas containing either earlyborn or lateborn neurons. Their classification was based on the relative fluorescence signal ratios of two incubated dyes (JF_552_/(JF_552_+JF_669_), which served as birthdate signatures. For macro-regions analysis of Fig. 4a, we approximately separated the OT into three thirds (roughly corresponding to a threshold fluorescence ratio of ≈0.3), being the central region corresponding to mature neurons. After motion correction, GCaMP transients within these macro-regions were integrated and converted to ΔF/F₀ signals, where F₀ was defined as the 20th quantile of the full intensity distribution. (ii) For single cell analysis, GCaMP fluorescence was extracted from individual neurons identified using CellPose^77^ (cyto2 model applied to JF dye fluorescence) with manual refinement of ROIs in ImageJ. After background subtraction and motion correction, GCaMP transients were converted to ΔF/F₀ signals, with F₀ calculated as the time-averaged intensity of each trace. For each neuron, the unmixed ratio of the two incubated dyes was also computed from the same ROI. Peaks of ΔF/F₀ signals were defined as the maximum values within a 3 second time window starting at the onset of each visual stimulus.

To compare between early and lateborn neuron activity, the spearman coefficient between the dye ratio and the ΔF/F, and for each timepoint (imaging frame) was calculated using scipy’s Spearman function (Fig. 4b–iii). We then plotted the spearman coefficient in function of time. Spearman coefficient (non-parametric test for two continuous variables): 1 = perfectly correlated, 0 = not correlated, −1 = anticorrelation. Significant results are given in red.

For the DSI analysis, only neurons which responded to at least one of the gratings were selected. The response amplitudes were defined as the max ΔF/F over the 10 frames following the stimulus onset. Responsive neurons were defined when response amplitudes were larger than 4 times the standard deviation. (evaluated in the 20 frames before stimulus onset). For each of the eight moving bars stimulus, a vector was defined by its angle corresponding to the bar direction and its response amplitude. The DSI was calculated as the magnitude of the vectorial sum of these eight vectors, normalized by the sum of their individual magnitudes (Fig. 4b–iv). We separated neurons into two equal sized groups (according to the dye ratio) and plotted the distribution of preferred directions (Supplementary Fig. 6c).

#### Spontaneous Calcium and Voltage Imaging analysis

WHaloCaMP fluorescence transients from spinning disk recordings were extracted by randomly selecting 50 cells for each birthdate-labeled population (Fig. 6a). Fluorescence raw traces were integrated, and, after detrending and smoothing, ΔF/F was calculated. Spontaneous activity for each cell was estimated as the integral of the ΔF/F trace above baseline. Importantly, previous characterization^49^ demonstrated similar sensitivity of WHaloCaMP transients when using either JF_552_ or JF_669_ dyes, ensuring that observed differences in fluorescence reflect genuine variations in neuronal activity between the two populations. Additionally, the high selectivity in the excitation and detection of each dye minimizes cross-talk, avoiding contamination of the functional signal due to dual-color labeling.

Voltron2_JF552_ and Voltron2_JF669_ fluorescent transients obtained from light-sheet imaging were extracted for each birthdate-labeled population (Fig. 6b) in the zebrafish olfactory epithelium. Fluorescent transients were integrated over ROIs covering individual cell bodies, and used to estimate ΔF/F, after detrending and background subtraction. Cells exhibiting some narrow ΔF/F peaks above noise level, corresponding to spikes activity, were declared active. Spiking frequency was estimated by manually counting spikes from active cells.

#### Analysis of longitudinal functional imaging of the hindbrain

##### Image processing

The GCaMP channel of the functional imaging data was used as a source to determine the mismatch between the odd and even scan lines and the lateral shift of the sample in each plane by phase cross correlation (the phase_cross_correlation function from scikit-image library). Then these corrections were applied to all the channels, and a high signal-to-noise ratio volume was generated for each channel by averaging across all the time points. Bleed-through of GCaMP signal to the JF_585_ and JF_646_ channels was estimated based on the voxels within the top ten percentile GCaMP signal with a linear regression. The JF_585_ and JF_646_ channels were subtracted with the bleed-through estimated voxel-by-voxel and then the subtracted volume were normalized between the median and 99.99 percentile voxel values as described above. The summed image of the two JF dye signals was then up-sampled at two times higher resolution and fed to the nuclei detection algorithm based on local contrast^78^. Then, the JF_585_ and JF_646_ signals from each of the detected nuclei were extracted to define the two neuronal age groups based on the vector angle defined by the normalized JF_585_ and JF_646_ signals, $\text{arctan}\left(\frac{\langle S_{\text{JF}646}\rangle_{\text{norm}}}{\langle S_{\text{JF}585}\rangle_{\text{norm}}}\right)$. As a first step, the local peaks detected in the skin with high autofluorescence in the JF_585_ channel were removed by applying Gaussian Mixture Model (GMM) on the vector angle using GaussianMixture from scikit-learn. This group was operationally defined as one of the three components detected by GMM with the lowest vector angle since the skin autofluorescence was mostly absent in the JF_646_ channel. Subsequently, remaining local peaks from neuronal nuclei were divided into two neuronal age groups using sigma clipping. This procedure is motivated by our observation that the second age group corresponds to the long tail on the higher angle. Thus, the second age group was defined as the group with the angle higher than three standard deviations away from the Gaussian distribution detected by sigma clipping. The ΔF/F0 of GCaMP signal from individual neurons was calculated by dividing raw fluorescence signal from each identified nucleus with the baseline estimated as a twenty-percentile value within a 60-second rolling window and used in the following regression and clustering analyses.

#### Analysis of locomotor activity

EMG traces are processed to detect stimulus-induced escapes and spontaneous swims as follows. First, large transient deflections caused by the electrical stimuli were first identified by the find_peaks function from scipy library and the time windows dominated by the artifacts were blanked (typically −1.5 to +5 ms around the peak of the artifact). A slow decay component of the artifact after large transients was modeled by the interp1d function from scipy and subtracted from the trace. Then, the EMG traces from the left and the right sides of the axial muscles were processed as follows to generate a smoothed EMG amplitude estimate robust against noise. They were first z-score normalized, then their power in a local Gaussian Kernel window (8 ms at full-width at half maximum) was calculated, and its square root product was used as a robust EMG amplitude estimate. Then, the peaks corresponding to each burst of muscle activity were detected by the find_peaks function for the left and right EMG traces separately. To reject the peaks corresponding to the strong EMG bursts from the other side, the following procedure was taken. After merging the peaks from both sides, each pair of neighboring bursts were compared, and if the inter-peak interval was shorter than 6 ms, the smaller peak was excluded from further analysis. Then swim episodes were defined as a series of bursts whose interval stayed less than 70 ms. Instances of burst groupings with less than 4 bursts (corresponding to 2 left-right alternations) were not classified as swim episodes.

#### Regression analysis of locomotor activity

To map the neurons recruited during distinct types of locomotor activity, regression analysis was performed on ΔF/F0 of the GCaMP signal extracted from individual nuclei using regressors representing distinct swim events. In both developmental stages, escape events were defined based on the electrical shock that reliably induced escape behavior. In 48 hpf embryos, another regressor was constructed to capture the variability in the length of shock-induced swim episodes by treating each detected burst from EMG traces from both sides. In 96 hpf embryos, we constructed three regressors besides the escape regressor based on the expanded range of EMG burst amplitudes (Fig. 5c), similarly to^10^. Bursts from each EMG trace were classified as ‘weak’ or ‘strong’ using GMM with two components. Then, the ‘strong’ bursts from left and right EMG traces were treated as separate events based on our previous observation that distinct sets of neurons are recruited based on the side of strong locomotor activity. The ‘weak’ bursts from left and right EMG traces were combined together as in^10^ given the slow kinetics of jGCaMP7s relative to left-right alternating bursting activity. Regressors were constructed by convolving each of these locomotor event types with the GCaMP6s impulse response function given its similar kinetics to jGCaMP7s^79^, and then z-score standardized for each session. Neuronal ΔF/F0 response matrix (Y) was fitted with these standardized regressors (X) using the following linear model, Y= Xβ+ ϵ. The standardized coefficient (β) was estimated by ordinary least square and then T value for each neuron was calculated based on standardized coefficient (β) and residual (ϵ). Correction for multiple comparisons was done with the false discovery rate (FDR). The threshold for T maps was set at P_FDR_ <0.05. The neurons that meet thiscriteria for a given locomotor event were classified as active neurons. To statistically examine if neuronal age and subject age explained the variance in the proportion of active neurons in each group, we employed a generalized linear mixed model with a logit link function for binomial distribution using the lme4 package in R (https://cran.r-project.org/web/packages/lme4/index.html) as follows:

$$\text{Presence of active neuron}\sim \text{neuron\_age}*\text{subject\_age}+\left(1|\text{subject\_id}\right)+\left(1|\text{locomotor\_type}\right)$$

Besides subject identity, locomotor type was also treated as a random effect since 2 dpf and 4 dpf fish showed distinct locomotor behavior. After observing statistically significant effects of subject age and neuronal age and their interaction, post hoc Tukey tests were carried out using the emmeans package in R to correct for multiple comparisons.

#### Functional clustering of the hindbrain activity

The computed GCaMP ΔF/F0 from all the identified neurons were first z-score normalized over time and then subjected to Rastermap algorithm^48^ to sort them based on the similarity of their activity. Parameters were set as follows: n_PCs = 50, n_clusters = 100, locality = 0.1, time_lag_window = 0. For visualization, neuronal activity was averaged in bins of 50 neurons-the averages of these neurons are called ‘superneurons’. The age of ‘superneuron’ was computed by averaging the age of individual neurons in each superneuron. The sorted neurons were subdivided into eighteen functional groups based on their position in the Rastermap sorting. Neurons in each functional groups were divided into two neuronal age groups and visualized in top-down view.

#### Analysis of all-optical experiment

For the all-optical experiments in Fig. 6c, fluorescence signals were integrated over circular ROIs (13–15 μm for mammalian neurons and 8 μm for zebrafish neurons) centered on individual targeted or non-targeted somata. Photostimulation artifacts were semi-automatically detected and removed using a GUI-based MATLAB script. Percentage changes in fluorescence were calculated as ΔF/F = (F−F_0_)/F_0_, where F_0_ is the baseline fluorescence level. F_0_ was manually defined within the MATLAB GUI as the average fluorescence over ~5 s before holographic stimulation, enabling exclusion of spontaneous activity effects.

## Supplementary Material

1

## Data Availability

All data are available in the main text or the supplementary materials.

## References

[1] Tau G. Z. & Peterson B. S. Normal Development of Brain Circuits. Neuropsychopharmacology 35, 147–168 (2010). 19794405 10.1038/npp.2009.115 PMC3055433

[2] Tosches M. A. Developmental and genetic mechanisms of neural circuit evolution. Dev. Biol. 431, 16–25 (2017). 28645748 10.1016/j.ydbio.2017.06.016

[3] Baier H. & Scott E. K. The Visual Systems of Zebrafish. Annu. Rev. Neurosci. 47, 255–276 (2024). 38663429 10.1146/annurev-neuro-111020-104854

[4] Luo L. Architectures of neuronal circuits. Science (80-.). 373, (2021). 10.1126/science.abg7285 PMC8916593 34516844

[5] Osseward P. J. Conserved genetic signatures parcellate cardinal spinal neuron classes into local and projection subsets. Science (80-.). 372, 385–393 (2021). 10.1126/science.abe0690 PMC8612134 33888637

[6] Mathews E. A. A Distinctive layering pattern of mouse dentate granule cells is generated by developmental and adult neurogenesis. J. Comp. Neurol. 518, 4479–4490 (2010). 20886617 10.1002/cne.22489 PMC2997649

[7] Espinosa J. S. & Luo L. Timing Neurogenesis and Differentiation: Insights from Quantitative Clonal Analyses of Cerebellar Granule Cells. J. Neurosci. 28, 2301–2312 (2008). 18322077 10.1523/JNEUROSCI.5157-07.2008 PMC2586640

[8] Marachlian E., Avitan L., Goodhill G. J. & Sumbre G. Principles of Functional Circuit Connectivity: Insights From Spontaneous Activity in the Zebrafish Optic Tectum. Front. Neural Circuits 12, (2018). 10.3389/fncir.2018.00046 PMC6021757 29977193

[9] Kinkhabwala A. A structural and functional ground plan for neurons in the hindbrain of zebrafish. Proc. Natl. Acad. Sci. 108, 1164–1169 (2011). 21199947 10.1073/pnas.1012185108 PMC3024665

[10] Pujala A. & Koyama M. Chronology-based architecture of descending circuits that underlie the development of locomotor repertoire after birth. Elife 8, (2019). 10.7554/eLife.42135 PMC6449084 30801247

[11] Goldblatt D. Neuronal birthdate reveals topography in a vestibular brainstem circuit for gaze stabilization. Curr. Biol. 33, 1265–1281.e7 (2023). 36924768 10.1016/j.cub.2023.02.048 PMC10089979

[12] Boulanger-Weill J. & Sumbre G. Functional integration of newborn neurons in the zebrafish optic tectum. Front. Cell Dev. Biol. 7, 1–8 (2019). 31058148 10.3389/fcell.2019.00057 PMC6477100

[13] Boulanger-Weill J. Functional Interactions between Newborn and Mature Neurons Leading to Integration into Established Neuronal Circuits. Curr. Biol. 27, 1707–1720.e5 (2017). 28578928 10.1016/j.cub.2017.05.029 PMC5483231

[14] Zhao X. & van Praag H. Steps towards standardized quantification of adult neurogenesis. Nat. Commun. 11, 1–10 (2020). 32848155 10.1038/s41467-020-18046-y PMC7450090

[15] Eriksson P. S. Neurogenesis in the adult human hippocampus. Nat. Med. 4, 1313–1317 (1998). 9809557 10.1038/3305

[16] Chan U., Gautam D. & West A. E. Utilizing in vivo postnatal electroporation to study cerebellar granule neuron morphology and synapse development. J. Vis. Exp. 2021, (2021). 10.3791/62568 PMC10476226 34180898

[17] Yang C., Shitamukai A., Yang S. & Kawaguchi A. Advanced Techniques Using In Vivo Electroporation to Study the Molecular Mechanisms of Cerebral Development Disorders. Int. J. Mol. Sci. 24, (2023). 10.3390/ijms241814128 PMC10531473 37762431

[18] Weber T. & Köster R. Genetic tools for multicolor imaging in zebrafish larvae. Methods 62, 279–291 (2013). 23886907 10.1016/j.ymeth.2013.07.028

[19] Livet J. Transgenic strategies for combinatorial expression of fluorescent proteins in the nervous system. Nature 450, 56–62 (2007). 17972876 10.1038/nature06293

[20] Bourgeois D. & Adam V. Reversible photoswitching in fluorescent proteins: A mechanistic view. IUBMB Life 64, 482–491 (2012). 22535712 10.1002/iub.1023

[21] Garcia-Marques J. A programmable sequence of reporters for lineage analysis. Nat. Neurosci. 23, 1618–1628 (2020). 32719561 10.1038/s41593-020-0676-9

[22] Espinosa-Medina I. TEMPO enables sequential genetic labeling and manipulation of vertebrate cell lineages. Neuron 111, 345–361.e10 (2023). 36417906 10.1016/j.neuron.2022.10.035

[23] Emiliani V. Optogenetics for light control of biological systems. Nat. Rev. Methods Prim. 2, 55 (2022). 10.1038/s43586-022-00136-4 PMC10627578 37933248

[24] Grienberger C., Giovannucci A., Zeiger W. & Portera-Cailliau C. Two-photon calcium imaging of neuronal activity. Nat. Rev. Methods Prim. 2, 67 (2022). 10.1038/s43586-022-00147-1 PMC10732251 38124998

[25] Chen I. W. In Vivo submillisecond two-photon optogenetics with temporally focused patterned light. J. Neurosci. 39, 3484–3497 (2019). 30833505 10.1523/JNEUROSCI.1785-18.2018 PMC6495136

[26] Deo C. The HaloTag as a general scaffold for far-red tunable chemigenetic indicators. Nat. Chem. Biol. 17, 718–723 (2021). 33795886 10.1038/s41589-021-00775-w

[27] Grimm J. B. A general method to fine-tune fluorophores for live-cell and in vivo imaging. Nat. Methods 14, 987–994 (2017). 28869757 10.1038/nmeth.4403 PMC5621985

[28] Los G. V HaloTag: A Novel Protein Labeling Technology for Cell Imaging and Protein Analysis. ACS Chem. Biol. 3, 373–382 (2008). 18533659 10.1021/cb800025k

[29] Grimm J. B. Bright photoactivatable fluorophores for single-molecule imaging. Nat. Methods 13, 985–988 (2016). 27776112 10.1038/nmeth.4034

[30] Mohar B. DELTA: a method for brain-wide measurement of synaptic protein turnover reveals localized plasticity during learning. Nat. Neurosci. (2025) doi:10.1038/s41593-025-01923-4. PMC12081306 40164741

[31] Kim D. EPSILON: a method for pulse-chase labeling to probe synaptic AMPAR exocytosis during memory formation. Nat. Neurosci. (2025) doi:10.1038/s41593-025-01922-5. 40164742

[32] Kuriya K. Direct visualization of replication dynamics in early zebrafish embryos. Biosci. Biotechnol. Biochem. 80, 945–948 (2016). 26923175 10.1080/09168451.2016.1141039

[33] Kim C.-H. Zebrafish elav/HuC homologue as a very early neuronal marker. Neurosci. Lett. 216, 109–112 (1996). 8904795 10.1016/0304-3940(96)13021-4

[34] Park H.-C. Analysis of Upstream Elements in the HuC Promoter Leads to the Establishment of Transgenic Zebrafish with Fluorescent Neurons. Dev. Biol. 227, 279–293 (2000). 11071755 10.1006/dbio.2000.9898

[35] Park H.-C. Structural comparison of zebrafish Elav/Hu and their differential expressions during neurogenesis. Neurosci. Lett. 279, 81–84 (2000). 10674626 10.1016/s0304-3940(99)00940-4

[36] Marquart G. D. High-precision registration between zebrafish brain atlases using symmetric diffeomorphic normalization. Gigascience 6, (2017). 10.1093/gigascience/gix056 PMC5597853 28873968

[37] Menelaou E., VanDunk C. & McLean D. L. Differences in the morphology of spinal V2a neurons reflect their recruitment order during swimming in larval zebrafish. J. Comp. Neurol. 522, 1232–1248 (2014). 24114934 10.1002/cne.23465 PMC4692166

[38] Meyer M. P. & Smith S. J. Evidence from *In Vivo* Imaging That Synaptogenesis Guides the Growth and Branching of Axonal Arbors by Two Distinct Mechanisms. J. Neurosci. 26, 3604–3614 (2006). 16571769 10.1523/JNEUROSCI.0223-06.2006 PMC6673851

[39] Messier J. E., Chen H., Cai Z.-L. & Xue M. Targeting light-gated chloride channels to neuronal somatodendritic domain reduces their excitatory effect in the axon. Elife 7, (2018). 10.7554/eLife.38506 PMC6130974 30091701

[40] Lim S. T., Antonucci D. E., Scannevin R. H. & Trimmer J. S. A Novel Targeting Signal for Proximal Clustering of the Kv2.1 K+ Channel in Hippocampal Neurons. Neuron 25, 385–397 (2000). 10719893 10.1016/s0896-6273(00)80902-2

[41] Forli A. Two-photon bidirectional control and imaging of neuronal excitability with high spatial resolution in vivo. Cell Rep. 22, 3087–3098 (2018). 29539433 10.1016/j.celrep.2018.02.063 PMC5863087

[42] Niell C. M. & Smith S. J. Development of Visual Response Properties in the Zebrafish Tectum. 45, 941–951 (2005). 10.1016/j.neuron.2005.01.047 15797554

[43] Gabriel J. P., Trivedi C. A., Maurer C. M., Ryu S. & Bollmann J. H. Layer-Specific Targeting of Direction-Selective Neurons in the Zebrafish Optic Tectum. Neuron 76, 1147–1160 (2012). 23259950 10.1016/j.neuron.2012.12.003

[44] Barker A. J., Helmbrecht T. O., Grob A. A. & desplan H. Functional, molecular and morphological heterogeneity of superficial interneurons in the larval zebrafish tectum. 2159–2175 (2021) doi:10.1002/cne.25082. 33278028

[45] Del Bene F. Filtering of visual information in the tectum by an identified neural circuit. Science (80-.). 330, 669–673 (2010). 10.1126/science.1192949 PMC3243732 21030657

[46] Förster D. Retinotectal circuitry of larval zebrafish is adapted to detection and pursuit of prey. Elife 9, 1–26 (2020). 10.7554/eLife.58596 PMC7550190 33044168

[47] McLean D. L. & Fetcho J. R. Spinal Interneurons Differentiate Sequentially from Those Driving the Fastest Swimming Movements in Larval Zebrafish to Those Driving the Slowest Ones. J. Neurosci. 29, 13566–13577 (2009). 19864569 10.1523/JNEUROSCI.3277-09.2009 PMC2796107

[48] Stringer C. Rastermap: a discovery method for neural population recordings. Nat. Neurosci. 28, 201–212 (2025). 39414974 10.1038/s41593-024-01783-4 PMC11706777

[49] Farrants H. A modular chemigenetic calcium indicator for multiplexed in vivo functional imaging. Nat. Methods 21, 1916–1925 (2024). 39304767 10.1038/s41592-024-02411-6 PMC11466818

[50] Abdelfattah A. S. Sensitivity optimization of a rhodopsin-based fluorescent voltage indicator. Neuron 111, 1547–1563.e9 (2023). 37015225 10.1016/j.neuron.2023.03.009 PMC10280807

[51] Emiliani V., Cohen A. E., Deisseroth K. & Häusser M. All-optical interrogation of neural circuits. J. Neurosci. 35, 13917–13926 (2015). 26468193 10.1523/JNEUROSCI.2916-15.2015 PMC4604230

[52] Deisseroth K. Optogenetics: 10 years of microbial opsins in neuroscience. Nat. Neurosci. 18, 1213–1225 (2015). 26308982 10.1038/nn.4091 PMC4790845

[53] Inoue T. & Krumlauf R. An impulse to the brain—using in vivo electroporation. Nat. Neurosci. 4,1156–1158 (2001). 11687822 10.1038/nn1101-1156

[54] dal Maschio M. High-performance and site-directed in utero electroporation by a triple-electrode probe. Nat. Commun. 3, 960 (2012). 22805567 10.1038/ncomms1961 PMC5972006

[55] LUO L. Fly MARCM and mouse MADM: Genetic methods of labeling and manipulating single neurons. Brain Res. Rev. 55, 220–227 (2007). 17408568 10.1016/j.brainresrev.2007.01.012 PMC2096471

[56] Yu H.-H., Chen C.-H., Shi L., Huang Y. & Lee T. Twin-spot MARCM to reveal the developmental origin and identity of neurons. Nat. Neurosci. 12, 947–953 (2009). 19525942 10.1038/nn.2345 PMC2701974

[57] Binns T. C. Rational Design of Bioavailable Photosensitizers for Manipulation and Imaging ofBiological Systems. Cell Chem. Biol. 27, 1063–1072.e7 (2020). 32698018 10.1016/j.chembiol.2020.07.001 PMC7483975

[58] Wu J. Kilohertz two-photon fluorescence microscopy imaging of neural activity in vivo. Nat. Methods 17, 287–290 (2020). 32123392 10.1038/s41592-020-0762-7 PMC7199528

[59] Liu Z. Sustained deep-tissue voltage recording using a fast indicator evolved for two-photon microscopy. Cell 185, 3408–3425.e29 (2022). 35985322 10.1016/j.cell.2022.07.013 PMC9563101

[60] Chang Y. & Dickinson D. J. Non-invasive chimeric HaloTag labeling to study clustering and diffusion of membrane proteins. STAR Protoc. 3, 101857 (2022). 36595905 10.1016/j.xpro.2022.101857 PMC9676207

[61] Couturier L. HaloTag-based reporters for sparse labeling and cell tracking. Fly (Austin). 16, 360–366 (2022). 36323649 10.1080/19336934.2022.2142460 PMC9635558

[62] Grimm J. B. A general method to improve fluorophores for live-cell and single-molecule microscopy. Nat. Methods 12, 244–250 (2015). 25599551 10.1038/nmeth.3256 PMC4344395

[63] Grimm J. B. A general method to optimize and functionalize red-shifted rhodamine dyes. Nat. Methods 17, 815–821 (2020). 32719532 10.1038/s41592-020-0909-6 PMC7396317

[64] Kimmel C. B., Ballard W. W., Kimmel S. R., Ullmann B. & Schilling T. F. Stages of embryonic development of the zebrafish. Dev. Dyn. 203, 253–310 (1995). 8589427 10.1002/aja.1002030302

[65] Spikol E. D., Cheng J., Macurak M., Subedi A. & Halpern M. E. Genetically defined nucleus incertus neurons differ in connectivity and function. Elife 12, (2024). 10.7554/eLife.89516 PMC11142643 38819436

[66] Bongiovanni C. BMP7 promotes cardiomyocyte regeneration in zebrafish and adult mice. Cell Rep. 43, 114162 (2024). 38678558 10.1016/j.celrep.2024.114162

[67] Dunn T. W. Brain-wide mapping of neural activity controlling zebrafish exploratory locomotion. Elife 5, (2016). 10.7554/eLife.12741 PMC4841782 27003593

[68] Bae Y.-K. Anatomy of zebrafish cerebellum and screen for mutations affecting its development. Dev. Biol. 330, 406–426 (2009). 19371731 10.1016/j.ydbio.2009.04.013

[69] Abdelfattah A. S. Bright and photostable chemigenetic indicators for extended in vivo voltageimaging. Science (80-.). 365, 699–704 (2019). 10.1126/science.aav6416 31371562

[70] Granato M. Genes controlling and mediating locomotion behavior of the zebrafish embryo and larva. Development 123, 399–413 (1996). 9007258 10.1242/dev.123.1.399

[71] White R. M. Transparent Adult Zebrafish as a Tool for In Vivo Transplantation Analysis. Cell Stem Cell 2, 183–189 (2008). 18371439 10.1016/j.stem.2007.11.002 PMC2292119

[72] Preibisch S., Saalfeld S. & Tomancak P. Globally optimal stitching of tiled 3D microscopic image acquisitions. Bioinformatics 25, 1463–1465 (2009). 19346324 10.1093/bioinformatics/btp184 PMC2682522

[73] Lindsey B. W., Douek A. M., Loosli F. & Kaslin J. A Whole Brain Staining, Embedding, and Clearing Pipeline for Adult Zebrafish to Visualize Cell Proliferation and Morphology in 3-Dimensions. Front. Neurosci. 11, (2018). 10.3389/fnins.2017.00750 PMC5776138 29386991

[74] Koyama M., Kinkhabwala A., Satou C., Higashijima S. & Fetcho J. Mapping a sensory-motor network onto a structural and functional ground plan in the hindbrain. Proc. Natl. Acad. Sci. 108, 1170–1175 (2011). 21199937 10.1073/pnas.1012189108 PMC3024692

[75] Faini G. Ultrafast light targeting for high-throughput precise control of neuronal networks. Nat. Commun. 14, 1888 (2023). 37019891 10.1038/s41467-023-37416-w PMC10074378

[76] Papagiakoumou E., Ronzitti E. & Emiliani V. Scanless two-photon excitation with temporal focusing. Nat. Methods 17, 571–581 (2020). 32284609 10.1038/s41592-020-0795-y

[77] Stringer C., Wang T., Michaelos M. & Pachitariu M. Cellpose: a generalist algorithm for cellular segmentation. Nat. Methods 18, 100–106 (2021). 33318659 10.1038/s41592-020-01018-x

[78] Kawashima T., Zwart M. F., Yang C.-T., Mensh B. D. & Ahrens M. B. The Serotonergic System Tracks the Outcomes of Actions to Mediate Short-Term Motor Learning. Cell 167, 933–946.e20 (2016). 27881303 10.1016/j.cell.2016.09.055

[79] Zhang Y. Fast and sensitive GCaMP calcium indicators for imaging neural populations. Nature 615, 884–891 (2023). 36922596 10.1038/s41586-023-05828-9 PMC10060165

