# Supplementary material for: CHLOK: a chemigenetic multicolor labeling system to visualize neuronal birthdate and circuit integration
Source: Res Sq. 2025 Jul 8:rs.3.rs-7039578. Preprint. [Version 1] doi: 10.21203/rs.3.rs-7039578/v1 (PMC12265168; doi:10.21203/rs.3.rs-7039578/v1)
Supplement: 1 [file NIHPPRS7039578V1-supplement-1.pdf]

# Supplementary Figures:

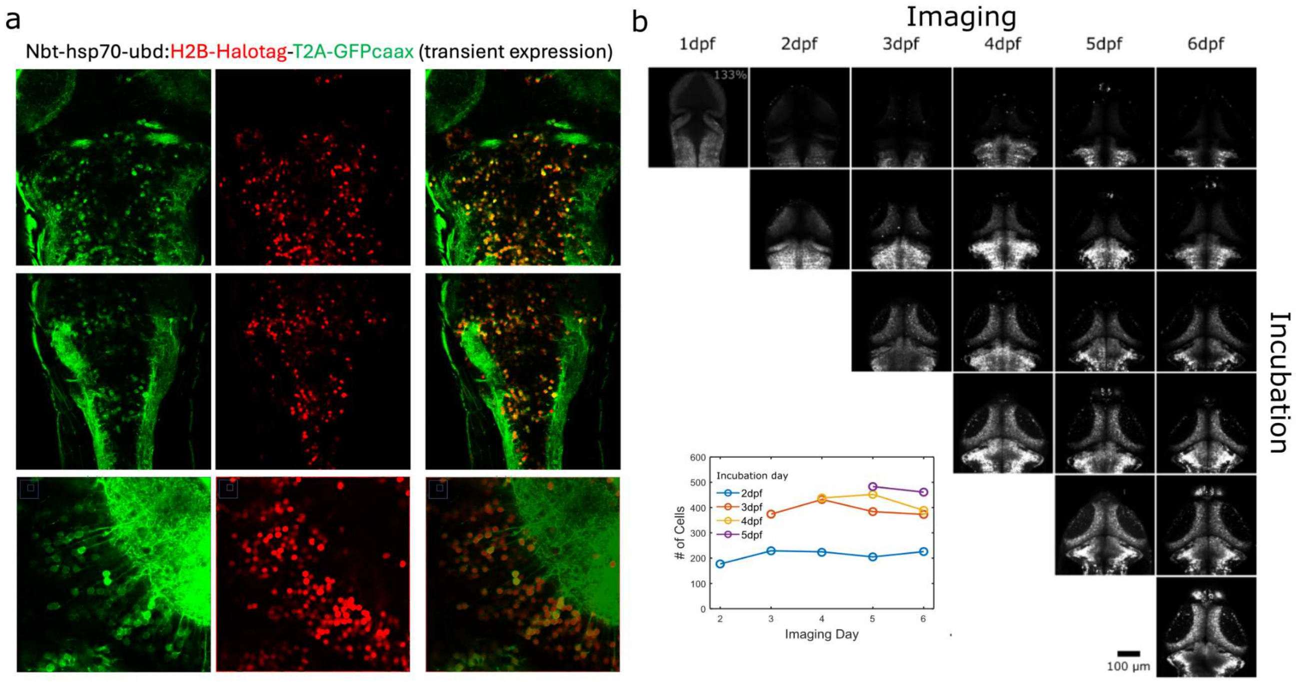


**Supplementary Figure 1. Efficient JF dye loading and neuronal labeling across the brain and developmental stages.** **a,** Images from the genetic fish line Tg (Xla.Tubb2-hsp70-ubc:H2B-HaloTag-T2A-GFPcaax), co-expressing GFP and Halotag. After incubation with $JF_{552}$, colocalization of the dye (red) and GFP (green) was evaluated to estimate the efficiency of the Halotag labelling (N = 3 larvae.97.43 ± 1.8 %). **b,** Set of confocal images from larvae labelled with a single dye at specific time points and imaged at different dpf. In the inset, we report the number of labeled cells for the same labeling timepoints but different age of imaging (N = 15 larvae).

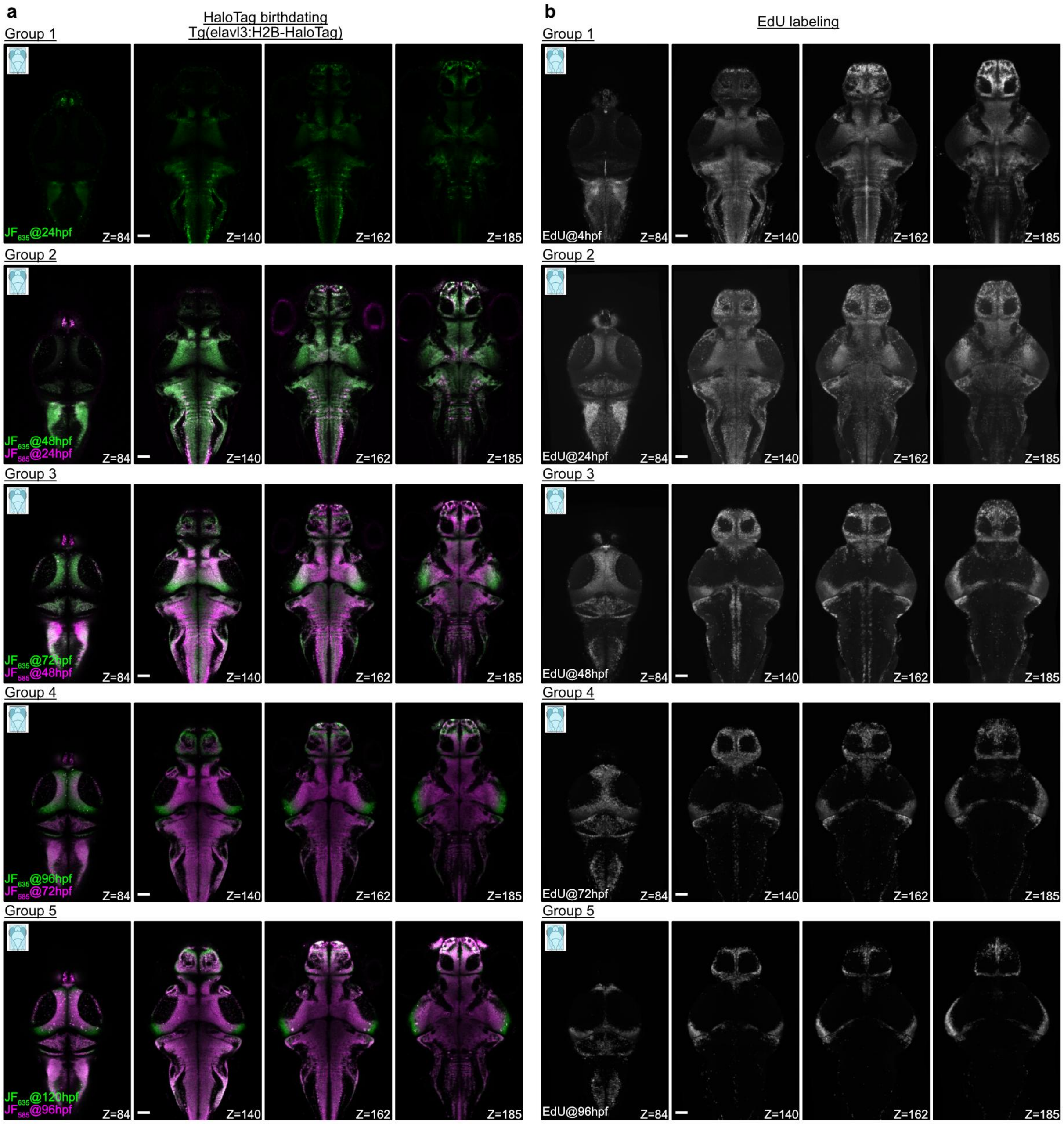


**Supplementary Figure 2. Individual age groups labeled by single-pulse HaloTag birthdating and EdU labeling. a,** Horizontal slices showing neurons birthdated with single-pulse HaloTag labeling for five-time windows. Group 1: 0-24 hpf; Group 2: 24-48 hpf; Group 3: 48-72 hpf; Group 4: 72-96 hpf; Group 5: 96-120 hpf. $JF_{585}$ labels the neurons before the time window of interest. $JF_{635}$ labels the neurons born during the time window of interest. The Z value in each horizontal slice indicates the dorsoventral position in the ZBB space. Each volume is a median of more than 4 samples. **b,** Horizontal slices showing the cells labeled by EdU introduced at 4 hpf (Group 1), 24 hpf (Group 2), 48 hpf (Group 3), 72 hpf (Group 4), and 96 hpf (Group 5). Horizontal slices are organized as in a. Each volume is a median of more than 4 samples. Scale bars, 50 μm.

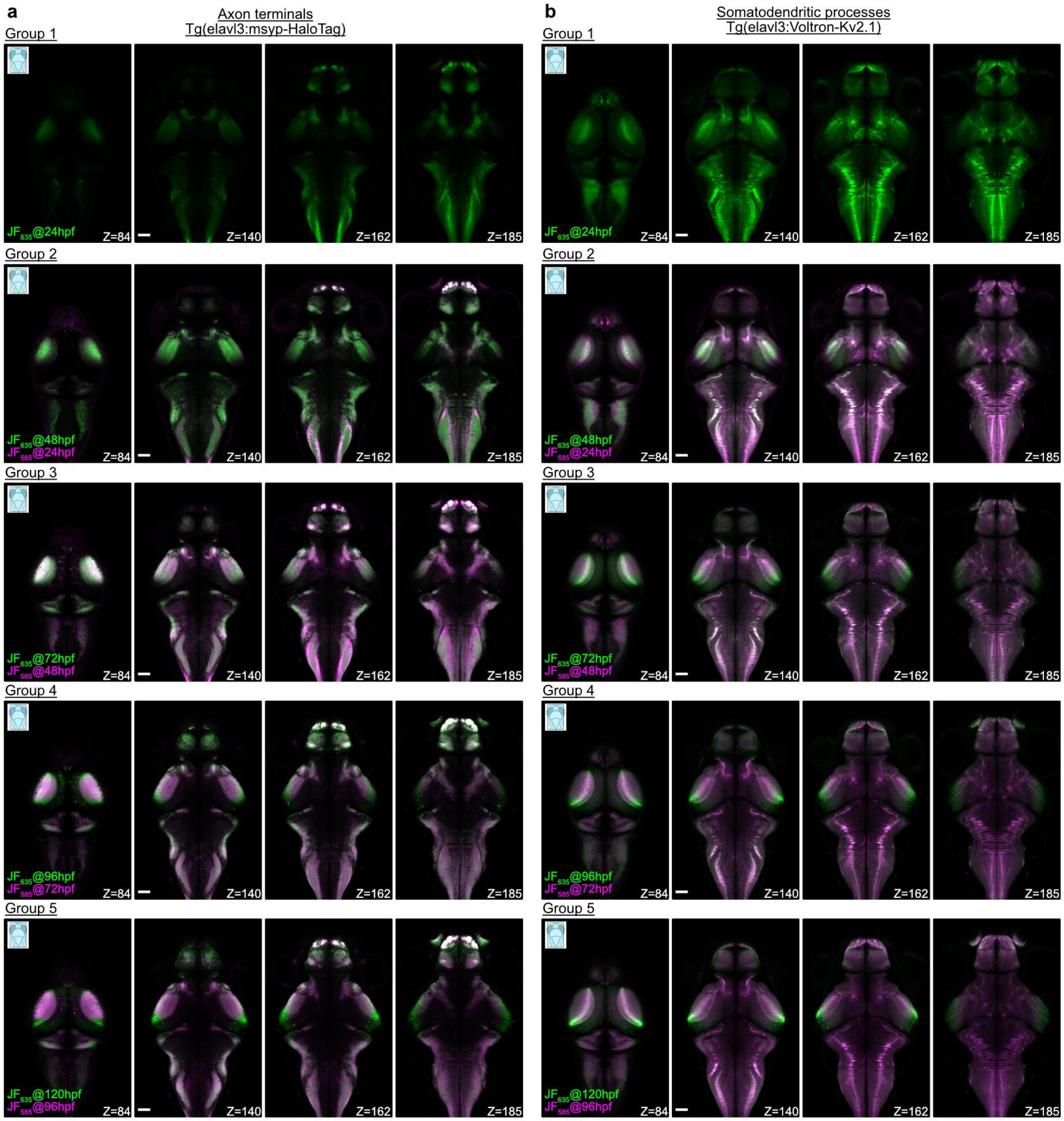


**Supplementary Figure 3. Subcellular processes visualized by single-pulse HaloTag birthdating. a,** Axon terminals visualized by single-pulse HaloTag birthdating. Group 1: 0-<24 hpf; Group 2: 24-48 hpf; Group 3: 48-72 hpf; Group 4: 72-96 hpf; Group 5: 96-120 hpf. $JF_{585}$ was loaded before the time window of interest. $JF_{635}$ was loaded at the end of the time window of interest. The Z value in each horizontal slice indicates the dorsoventral position in the ZBB space. Each volume is a median of more than 4 samples. **b,** Somatodendritic processes visualized by single-pulse HaloTag birthdating. Images are organized as in a. Scale bars, 50 µm.

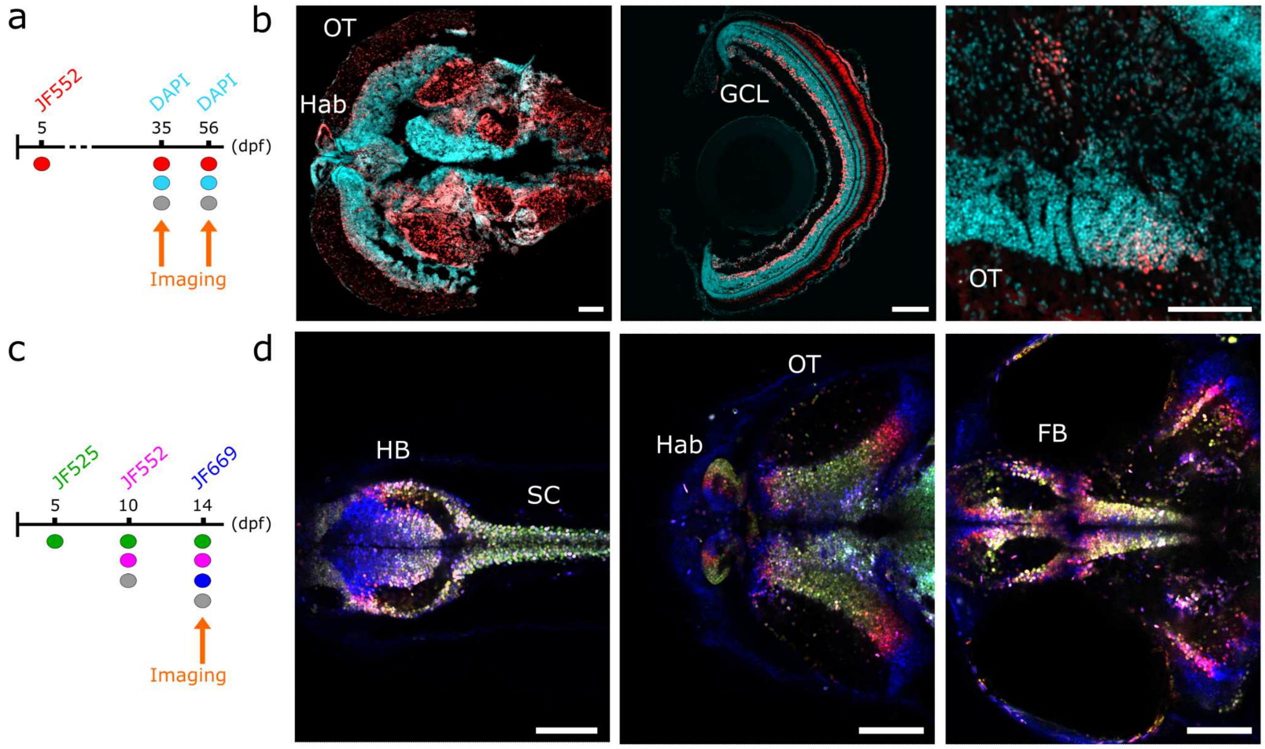


**Supplementary Figure 4. Single and multiple JF dyes labeling in juvenile stages. a,** Protocol of dye incubation and confocal imaging. The dye has been added at 5 dpf and tissue fixation was performed at 35 dpf or 56 dpf. **b,** Left, middle: Cryosections (16µm thickness) from a 5 weeks-old larva (35 dpf). Right: Cryosections (16µm thickness) from a 8 weeks-old larva (35 dpf). Different regions are shown: brain and retina. Red: $JF_{552}$ labeling, showing expression of H2B-Halotag. DAPI, in cyan stains neuronal soma (and not neuronal cells) born between 5dpf and the time of tissue fixation (35 or 56 dpf). **c,** Protocol of multilabelling and confocal imaging. Dyes have been added at 5 dpf ($JF_{525}$, green), 10 dpf ($JF_{552}$, magenta) and 14 dpf ($JF_{669}$, blue). Tissue fixation was performed at 14 dpf.

Scale bars: 100µm. OT: optic tectum. Hab: habenula. GCL: ganglion cell layer. HB: hindbrain. SC: spinal cord. FB: forebrain.

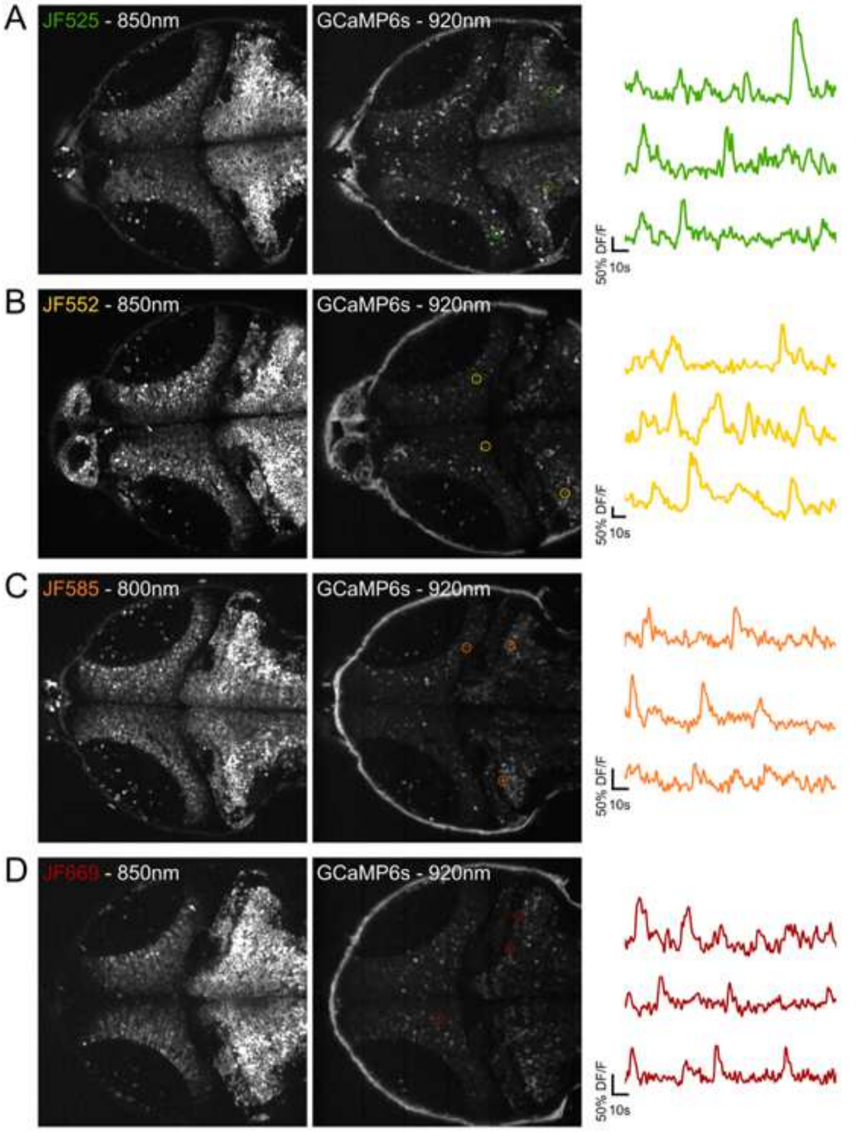


**Supplementary Figure 5. Spectral crosstalk of GCaMP and JF dyes.** Two photon images of the OT of larvae co-expressing Halotag (incubated with different JF dyes) and GCaMP6s. Imaging at 920 nm excitation wavelength allowed detection of spontaneous calcium transients (circles indicate the cells of interest; right: exemplary calcium traces).

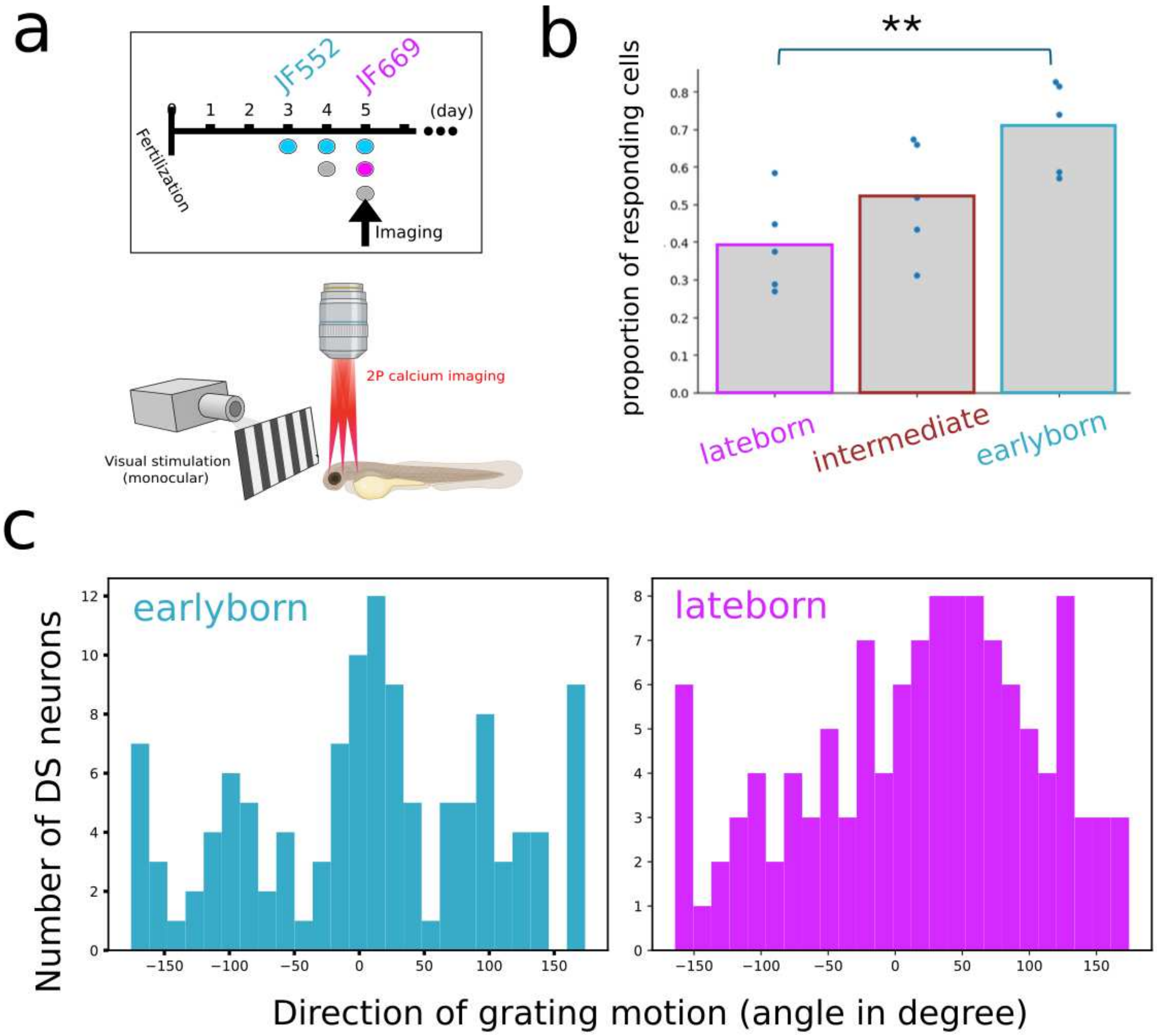


**Supplementary Figure 6. Visual activity of early- and lateborn neurons. a,** Experimental pipeline. Top: dual-color labeling in tg(Xla.Tubb2-hsp70-ubc:H2B-Halotag; elavl3:H2B-GCaMP6s) at 2 different time points during development (2 and 5dpf). Bottom: Schematic of the experimental setup, consisting in recording calcium activity under 2-photon illumination, while presenting a set of visual stimuli to the larvae, embedded in agar. **b,** Proportion of responsive neurons in three equal-sized groups based on dye ratio (lateborn, intermediate and earlyborn neurons), calculated as the average $\Delta F/F$ throughout all the visual stimulation epochs. *$p=0.01$, 1-way ANOVA. **c,** Distribution of preferred direction of moving for earlyborn (cyan) and lateborn (magenta) neurons.

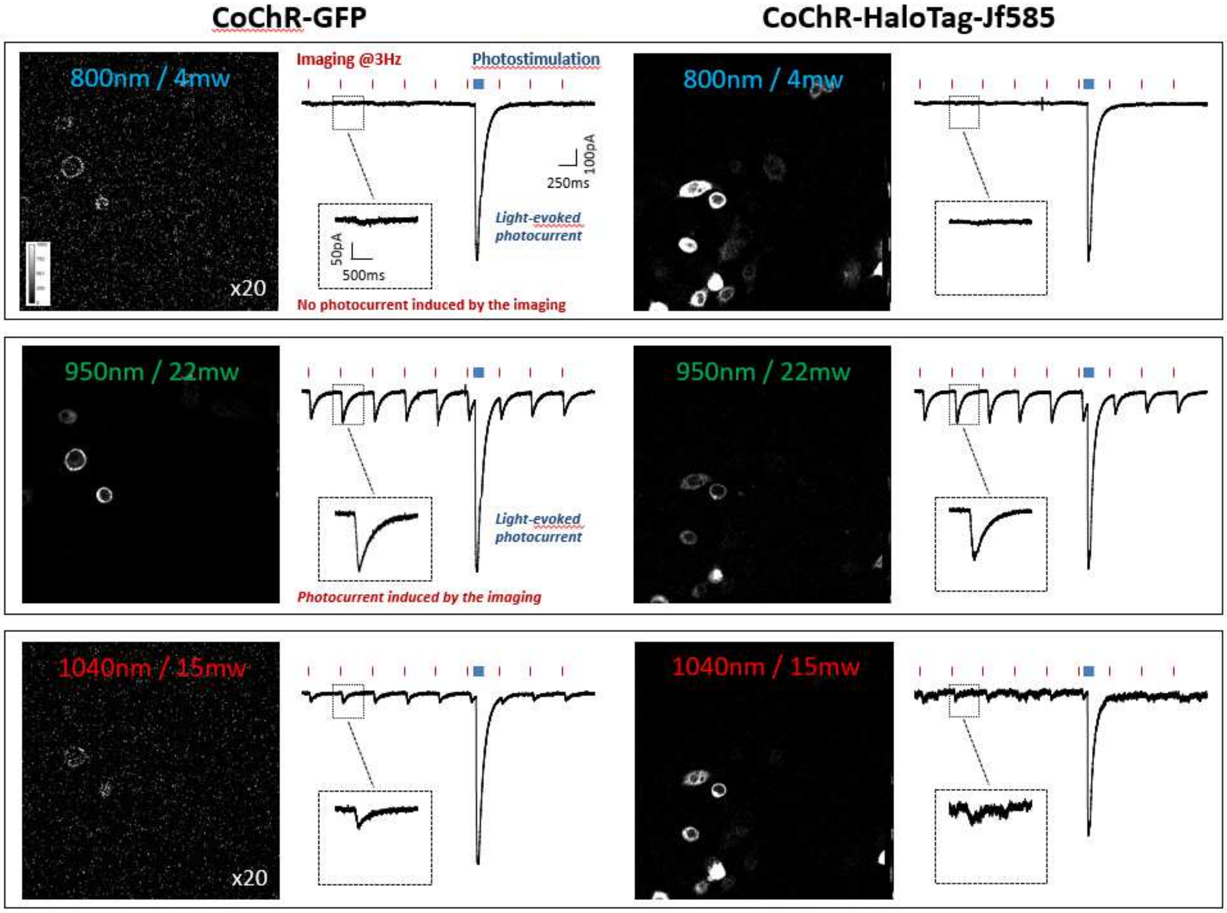


**Supplementary Figure 7. Imaging Induced opsin stimulation**. Images and measured photocurrents (patch clamp recording) from CHO cells expressing CoChR tagged with either GFP (left) or Halotag-$JF_{585}$ (right). Recordings are performed during 2P scanning imaging at different wavelengths and concurrent with holographic stimulation (blue tick). We observe how high contrast images of JF were achieved with low imaging power and using wavelength outside of the CoChR absorption peak (800 and 1040 nm), therefore reducing the artefactual photocurrent induced by the imaging laser. The same wavelengths are much less efficient in imaging GFP, that requires 950 nm and high imaging power to achieve comparable contrast images, inducing therefore high artefactual photocurrent from the imaging laser.

| | |
|---|---|
| Xla.Tubb2-hsp70-ubc:H2B-HaloTag; he1.1:CFP | stable line + plasmid |
| elavl3:H2B-HaloTag | stable line + plasmid |
| Xla.Tubb2-hsp70-ubc:H2B-HaloTag-T2A-GFPcaax; he1.1:CFP | stable line + plasmid |
| elavl3:sypb-HaloTag | stable line + plasmid |
| elavl3:voltron1-Kv2.1 | stable line + plasmid |
| elavl3:WhaloCaMP-eGFP | stable line + plasmid |
| 14UAS:CoChR-HaloTag; myl7:GFP | stable line + plasmid |
| Xla.Tubb2-hsp70-ubc:CoChR-HaloTag; he1.1:CFP | plasmid |
| Xla.Tubb2-hsp70-ubc:HaloTag; he1.1:CFP | plasmid |
| 14UAS:H2B-HaloTag; he1.1:CFP | plasmid |
| 14UAS:HaloTag; he1.1:CFP | plasmid |
| Xla.Tubb2-hsp70-ubc:H2B-HaloTag-linker-GFP | plasmid |
| 14UAS:CoChR-T2A-H2B-HaloTag ; he1.1:CFP | plasmid |
| Xla.Tubb2-hsp70-ub:CoChR-T2A-H2B-HaloTag; he1.1:CFP | plasmid |
| QUAS:voltron2; he1.1:CFP | plasmid |
| pAAV-SynCoChR-HaloTag | plasmid |

**Table 1: Plasmids and transgenic lines generated.**
